# A major QTL region associated with powdery mildew resistance in leaves and fruits of the reconstructed garden strawberry

**DOI:** 10.1007/s00122-025-04871-6

**Published:** 2025-04-07

**Authors:** Attiq ur Rehman, Jahn Davik, Petteri Karisto, Janne Kaseva, Saila Karhu, Marja Rantanen, Ismo Strandén, Timo Hytönen, Alan H. Schulman, Tuuli Haikonen

**Affiliations:** 1 Natural Resources Institute Finland (Luke), Helsinki, Finland; 2 Doctoral Program in Plant Sciences, University of Helsinki, Helsinki, Finland; 3 Luke Doctoral Program, Natural Resources Institute Finland (Luke), Helsinki, Finland; 4 Department of Molecular Plant Biology, Norwegian Institute of Bioeconomy Research, Ås, Norway; 5 Viikki Plant Science Centre, University of Helsinki, Helsinki, Finland; 6 Department of Agricultural Sciences, University of Helsinki, Helsinki, Finland; 7 Institute of Biotechnology, University of Helsinki, Helsinki, Finland

## Abstract

**Key message:**

Multiple QTLs for powdery mildew resistance were identified in a pre-breeding population derived from the octoploid progenitor species of garden strawberry, including a stable major novel factor on chromosome 3B.

**Abstract:**

Powdery mildew (PM), caused by the biotrophic fungal pathogen *Podosphaera aphanis*, poses an increasing threat to garden strawberry (*Fragaria* × *ananassa*) production worldwide. While a few commercial cultivars exhibit partial resistance, fungicide application remains essential for managing PM outbreaks. However, breeding offers a more sustainable approach for controlling PM. A better understanding of the genetics of resistance is required for informed breeding strategies, e.g. through identifying novel resistance factors derived from the progenitor species of garden strawberry, *F. chiloensis* and *F. virginiana*. We conducted genome-wide association (GWA) and multivariate analyses in a reconstructed (ReC) strawberry population to investigate PM resistance under natural infection. Leveraging multi-year field trial data and 20,779 single-nucleotide polymorphism markers, we identified a novel major quantitative trait locus (QTL) on chromosome 3B, designated as q.LPM.Rec-3B.2, that was consistently associated with high PM resistance in both leaves and fruits. Greenhouse validation with a subset of the ReC population confirmed that this QTL region was stable across field and greenhouse environments. Promising candidate genes for resistance, including two for *MLO* and one for *EXO70*, were identified within this major QTL. In addition, multi-locus GWA models and non-additive GWA revealed additional resistance QTLs on multiple chromosomes. Despite previous challenges in breeding for robust PM resistance due to its quantitative nature and complex genetic control, our results provide valuable insights into resistance-contributing QTL regions already existing in strawberry, novel wild-derived resistance QTLs not previously known, candidate genes, and pre-breeding germplasm carrying resistance traits as resources for future genome-informed breeding efforts.

**Supplementary Information:**

The online version contains supplementary material available at 10.1007/s00122-025-04871-6.

## Introduction

Powdery mildew (PM) is an economically important fungal disease in strawberry cultivation, potentially leading to unmarketable fruit and severe yield losses on untreated fields (Peries 1962). The causal agent of the disease is an obligate biotroph, *Podosphaera aphanis* (formerly known as *Sphaerotheca macularis*), which primarily infects the leaves, but also stolons, buds, flowers, and fruits of strawberry plants (Paulus 1990; Maas 1998; Braun et al. 2002). Considerable variability in PM resistance exists among commercial cultivars, with some exhibiting high susceptibility to PM, while resistant cultivars demonstrate varying degrees of resistance under fluctuating environmental conditions (Nelson 1996; Masny et al. 2016; Menzel 2022).

PM is prevalent in numerous strawberry-growing regions across the globe, both in open-field and high-tunnel cultivation systems (Nelson 1996). New plants from nurseries often serve as a source of infection in commercial fruit-growing areas (Jin 2016). Leaf symptoms are the most obvious indicators of the infection, while infected flowers and fruits result in poor fruit quality and even in arrested ripening. During the onset of PM infection, upwards curling of the leaf edges is the earliest visual symptom, sometimes accompanied with visible powdery mycelia. In severe cases, the infection may result in burning (necrotic lesions) at the leaf margins (Paulus 1990). Under ideal conditions for germination and colony establishment, conidial infection on host tissues can trigger disease symptoms within four days, initiating a new cycle of conidiation from white powdery mycelia formed on the abaxial leaf surface (Amsalem et al. 2006). Although high relative humidity is essential for the germination and release of conidia, conidial germination is hampered by the presence of free water (Peries 1962). The sexual ascospores that develop within cleistothecia on overwintering (persistent) plant foliage are considered to be a significant source of infection in early spring; therefore, eliminating old strawberry foliage helps to minimize epidemics (Gadoury et al. 2010). Management of PM predominantly relies on the application of foliar fungicides, but there is an increasing pressure to explore alternative methods to reduce agrochemical use and to overcome the reduction in fungicide sensitivity observed in field populations of *P. aphanis* (Palmer and Holmes 2021).

The garden strawberry, *F.* × *ananassa* Duchesne ex Rozier, is an allo-octoploid (2*n* = 8*x* = 56) commercial crop originally derived via hybridization between South American *F. chiloensis* Miller and North American *F. virginiana* Miller about 300 years ago in western Europe. Native populations of both progenitor species hold valuable diversity for important horticultural traits and resistance to various pests and diseases, including diverse sources of resistance to PM. This diversity has been extensively documented in numerous previous studies (Hancock et al. 1991, 2005, 2008; Luby et al. 2008; Cameron et al. 1993). A species reconstruction approach was proposed and implemented to combine pre-selected native clones of both *F. chiloensis* and *F. virginiana*, pooling a wide range of desirable alleles and traits via intraspecies, followed by interspecies, crosses (Hancock et al. 1993, 2010). By hybridizing several pre-improved selections of *F. chiloensis* and *F. virginiana*, we previously established a diverse multi-familial population of reconstructed (ReC) strawberry, which exhibits extensive genetic diversity for a wide range of horticultural traits (Rehman et al. 2024).

The previous studies focussing on garden strawberry germplasm have found moderate to high heritability for resistance to PM, depending on the genetic background and trial environment including open-field and protected conditions. Most studies have suggested complex genetics with mainly additive genetic effects for resistance (Simpson 1987; Davik and Honne 2005), but others emphasize more the non-additive effects (Daubeny 1961; Hsu et al 1969; Masny et al 2016). Nevertheless, the genetic architecture underlying PM resistance appears to be complex, as only a limited number of quantitative trait loci (QTLs) for leaf resistance have been identified thus far, and their effects have been inconsistent across environments and studies. To date, no QTLs specifically associated with fruit resistance have been reported (Cockerton et al. 2018; Sargent et al. 2019; Lynn et al. 2024). However, the previous studies in wild octoploid germplasm have found some accessions of *F. virginiana*, and in particular in its subspecies *virginiana*, to be quite resistant to PM, while *F. chiloensi*s ranges from partial to high susceptibility (Hancock et al. 2001; Kennedy et al. 2013). In this study, we evaluated the wild-derived ReC strawberry population for PM resistance in leaves and fruits in a multi-year field trial. Employing multi-model GWA, we identified a major QTL region in addition to multiple minor QTLs associated with PM resistance in leaves and fruits.

## Methods

### Plant material and field trial

A population of reconstructed strawberry (ReC) consisting of 319 individual F1 genotypes were evaluated in a field experiment at the Piikkiö Horticultural Research Station of Luke in Kaarina, southwestern Finland (60° 23′ N, 22° 33′ E). The ReC plant material consisted of F1-individuals from reconstruction crosses (Table S1) between pre-selected accessions of the progenitor species *F. virginiana* and *F. chiloensis*, as previously described in our earlier work that includes further details of the experimental layout and field management (Rehman et al. 2024). ReC seedlings were clonally propagated to eight plants per F1 individual. Plots containing four clonal plants from each genotype were planted in two replicate blocks, arranged as raised beds with plastic mulch and trickle irrigation lines. The field experiment was structured as 22 rows and 46 columns, with side borders planted with guard plants. Within each of the 22 rows, a control panel of commercial cultivars (four plants each) was incorporated, including ‘Lumotar’, ‘Ria’, ‘Korona’, ‘Polka’, and ‘Honeoye’. Furthermore, plots with additional controls including ‘Senga Sengana’ and ‘Kent’ were added in at least one row in both replicates.

### Greenhouse trial

A subset of 78 ReC individuals was observed for PM symptoms under greenhouse conditions. We randomly selected 10–12 individuals per progeny from seven progenies, namely PPPS_04, 05, 06, 07, 10, 11, and 13, and propagated them from runners taken from original seedling plants grown in the greenhouse. The tips were rooted in moist rockwool cubes in high humidity under mist. After the misting period, PM infections were allowed to proceed from PM-infected control plants. When plantlets were well-rooted, three clones of each ReC individual were placed in a hydroponic system in lettuce pots (PR 306, Vefi, Norway) in gutters containing circulated nutrient film (N:P:K + Ca of 17.6:2.4:12 + 10.5) as described previously (Haikonen et al. 2021). Two time-overlapping trials were set up one week apart. The ReC individuals of each progeny were divided into two trials, and plants were randomized into three replicate blocks per trial. The cultivar ‘Honeoye’ (*F.* × *ananassa*) was included as a control in both trials. During hydroponic growing, supplemental light was provided (18 h, 100 μmol m^−2^ s^−1^), and the temperature was maintained at + 22 °C day/ + 18 °C night.

### Leaf powdery mildew phenotyping

**Fig. 1:**
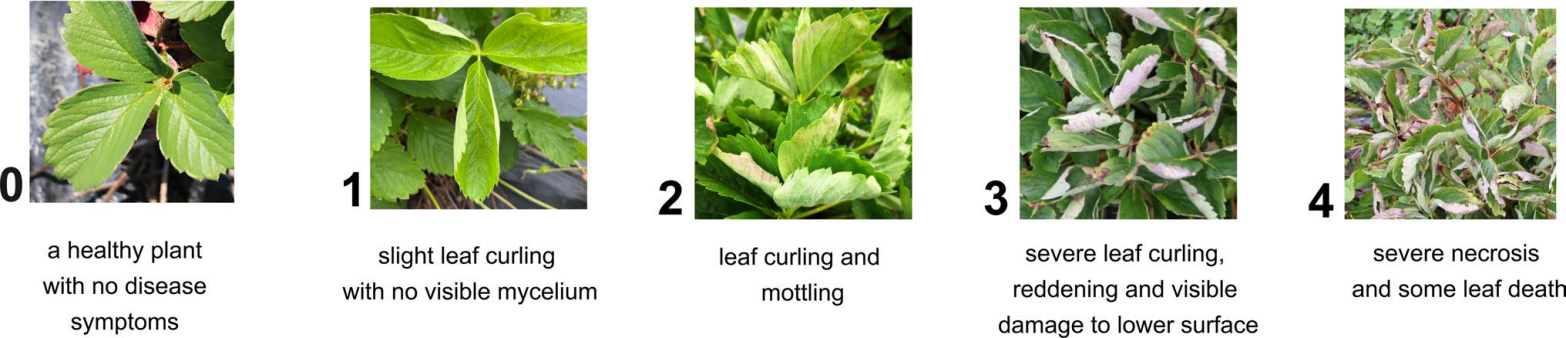
Disease severity scale applied to observe powdery mildew symptoms on strawberry leaves in the field trial; the severity level increased from 0 to 4

Leaf powdery mildew (LPM) severity was scored on per-plot basis for three seasons (2020, 2021, and 2022) in the field experiment. Plots were evaluated using the disease severity scale proposed by Simpson (1987) and illustrated in Fig. 1. Every plant in each plot was observed, and an average score was assigned to each plot, representing a single time point measurement. The field trial was scored on a biweekly basis at least five times per year between June and September to collect data on the PM disease progression. Plots with fewer than two surviving plants of the respective F1 individual genotype were excluded from observation to ensure standardized data quality for subsequent analysis.

In the greenhouse, LPM was observed using a three-point scale where: 0 denoted plants with healthy leaves; 1, leaves with mild symptoms; and 2, severely diseased foliage with upwards curling of young leaves and accumulation of mycelia on the leaflets. Observations were made three times, at the 1st, 2nd, and 3rd week after the start of the trials.

### Fruit powdery mildew phenotyping

The fruit powdery mildew (FPM) observations were recorded in the field experiment on a per-plot basis for two seasons (2021 and 2022) at the time of secondary berry ripening. Strawberry fruits were assessed using a four-point scale: 0 denoted no obvious mycelia; scores 1 and 2 showed rare or moderate mycelial coverage, respectively; and 3 indicated the presence of abundant mycelia on the fruits.

### Statistical analysis

Four aggregates of phenotypic data were derived from the raw data of field LPM disease severity for each season. These aggregates included: the mean, calculated as the average score per plot across all time points; the endpoint, representing the last measured score per plot; the AUDPC, the area under the disease progression curve; and the AUDPS, the area under the disease progression stairs, using all observed time points, taking the first time point as time 0, and following the formulas in Eqs. (1) and (2), respectively, using the *IdeTo* spreadsheet (Simko and Piepho 2012; Simko 2021).

1 $${\text{AUDPC}} = \mathop \sum \limits_{i = 1}^{n - 1} \left( {\frac{{y_{i} + y_{i + 1} }}{2}} \right) \times \left( {t_{i + 1} - t_{i} } \right),$$

where $y_{i}$ is the disease severity at the $i$ th time point, $t_{i}$ is the time at the $i$ th observation, and $n$ is the total number of observations.

2 $${\text{AUDPS}} = {\text{AUDPC}} + \left( {\frac{{y_{1} + y_{n} }}{2} + \frac{{t_{n} - t_{1} }}{n - 1}} \right),$$

where the terms $y_{1}$ and $y_{n}$ are the disease severity scores at $t_{1}$ and $t_{n}$ that refer to the initial and final week numbers of the time when the scores were taken, respectively. The $n$ represents the total number of observations. Trait-trait and trait-year correlations were inspected using *corrplot* R-package (Wei and Simko 2024).

All four LPM aggregates and the raw FPM scores were used as individual response variables in a linear mixed model, implemented in the *lmer* function of the *lme4* R-package (Bates et al. 2015). This approach was employed to estimate variance components by restricted maximum likelihood (REML) method and to compute best linear unbiased estimates (BLUEs) (Dataset S1). BLUEs for F1-individuals were calculated separately for each year using Eq. (3). The individual was treated as fixed, while the control cultivars, replicate, row, and column were included as random variables. Relevant design-based interactions of these variables with replicate, row, and column were also considered and included in the model as random factors (Eq. 3) if significant:

3 $$y_{ijklm} = \mu + G_{i} + C_{j} + R_{k} + {\text{row}}_{l} + {\text{col}}_{m} + G_{i} \times R_{k} + {\text{row}}_{l} \times {\text{col}}_{m} + G_{i} \times {\text{row}} _{l} + G_{i} \times {\text{col}}_{m} + \epsilon_{ijklm} ,$$

where $y_{ijklm}$ is the response variable (LPM aggregates or raw FPM score) for the individual $i$, *μ* is the overall mean, $G_{i}$ is the fixed effect of the individual $i$, $C_{j}$ is the random effect of the control cultivar $j$, $R_{k}$ is the random effect of the replicate $k$, ${\text{row}}_{l}$ is the random effect of the row $l$, ${\text{col}}_{m}$ is the random effect of the column $m$, $G_{i} \times R_{k}$ is the interaction between individual and replicate, ${\text{row}}_{l} \times {\text{col}}_{m}$ is the interaction term between row and column, while $G_{i} \times {\text{row}}_{l}$ and $G_{i} \times {\text{col}}_{m}$ are the interaction terms between individual and row, and individual and column, respectively. The residual error term is denoted by $\epsilon_{ijklm}$. Additionally, combined BLUEs (for the fixed effect of individuals) for all three years were computed by adding ‘year’ as a fixed factor in the same model.

The greenhouse dataset was analysed independently, and the *lmer* function was used to estimate REML variance components from mean scores using the positional information (rows and columns) of each individual in the trial (Eq. 4)

4 $$y_{ino} = \mu + G_{i} + R_{n} + C_{o} + \epsilon_{ino} ,$$

where $y_{ino}$ is the observed mean PM score for the individual $i$ at row position $n$ and column position $o$, $\mu$ is the overall mean, $G_{i}$ is the fixed effect of the individual $i$, $R_{n}$ is the random effect of the row position $n$, $C_{o}$ is the random effect of the column position $o$, whereas, $\epsilon_{ino}$ is the residual error.

All BLUEs for the individuals (*G*_*i*_) from Eqs. (3) and (4) were further used to create frequency distribution plots. These BLUEs were also used as inputs for GWA in the subsequent steps (Holland and Piepho 2024). Family-wise differences were inspected using ANOVA followed by Tukey’s HSD test in R (R Core Team 2020). Genetic variance components were estimated using REML, accounting for spatial variation within each year, and broad-sense heritability (H^2^) was computed using the *getHeritability* function in Spatial Analysis of Field Trials with Splines (*SpATS*) R-package (Rodríguez-Álvarez et al. 2018). The broad-sense heritability for combined years was calculated in R (R Core Team 2020) according to Eq. (5)

5 $$H^{2} = \frac{{\sigma_{{\text{G}}}^{2} }}{{\sigma_{{\text{G}}}^{2} + {{\sigma_{{\text{E}}}^{2} }}}},$$

where $\sigma_{{\text{G}}}^{2}$ and $\sigma_{{\text{E}}}^{2}$ refer to the genotypic and environmental variance, respectively $n_r$.

### Genotyping, population structure, and GWA using additive model

Genotyping of 319 ReC individuals was performed using the Axiom™ Strawberry FanaSNP 50k Genotyping Array (Hardigan et al. 2020), as described by Rehman et al. (2024). After quality control, which included filtering for call rate > 90%, and minor allele frequency (MAF) threshold of 1%, the final dataset comprised 298 individuals and 20,779 high-quality SNP markers for subsequent GWA analyses (Dataset S1). Physical positions and chromosome nomenclature of the SNPs are based on the subgenome- and haplotype-resolved Royal Royce V1 reference genome (Hardigan et al. 2021).

To identify marker-trait associations (MTAs), we employed one single-locus GWA method, the mixed linear model (MLM) (Yu et al. 2006), and two multi-locus methods: fixed and random model circulating probability unification (FarmCPU) (Liu et al. 2016) and Bayesian-information and linkage-disequilibrium iteratively nested keyway (BLINK) (Huang et al. 2019) using the genomic association and prediction integrated tool (GAPIT) v3.0 (Wang and Zhang 2021). To correct for the confounding effects of population structure, we performed principal component analysis (PCA) on the genotypic data using GAPIT, incorporating the first five principal components and the kinship matrix (VanRaden 2008) in our GWA. A Bonferroni threshold (− log10(*p*) = 5.61) was applied to report significant MTAs and to visualize GWA summary plots, QQ, and Manhattan plots using the *gwaspr* R-package (Wright 2024). A Miami plot was created by using the *hudson* package in R (Lucas et al. 2022). While a population structure analysis of the ReC population was reported previously (Rehman et al. 2024), we conducted an additional discriminant analysis of principal components to identify genetic clusters using the *adegenet* R-package (Jombart 2008). The identified clusters were then utilized to assess and visualize the phenotypic data.

In addition to single-trait GWA with different models, we inspected the SNP effects on leaf (LPM Endpoint_BLUEs) and fruit (FPM_BLUEs) powdery mildew using a multiple-trait model. We applied Bayesian multi-trait model with variable selection using spike-slab priors in the BGLR R-package (Pérez and de los Campos 2022). The calculations used 25,000 Markov chain Monte Carlo iterations, including a burn-in period of 5000 iterations. The model was

6 $${\mathbf{y}} = {\mathbf{1}}\mu + {\mathbf{X\beta }} + {{\varvec{\epsilon}}},$$

where $\mathbf{y}=[{\mathbf{y}}_{1},{\mathbf{y}}_{2},{\mathbf{y}}_{3},{\mathbf{y}}_{4}]$ is a vector of phenotypes (1, 2, 3, 4 for LPM Endpoint BLUEs 2021, LPM Endpoint BLUEs 2022, FPM BLUEs 2021, and FPM BLUEs 2022); ${\varvec{\upbeta}}=[{{\varvec{\upbeta}}}_{1},{{\varvec{\upbeta}}}_{2},{{\varvec{\upbeta}}}_{3},{{\varvec{\upbeta}}}_{4}]$ is a vector of marker effects; $\mathbf{X}$ is an $n\times m$ matrix of marker covariates (or genotype counts), *n* = 239 and *m* = 20,779; and ${\varvec{\epsilon}}=[{{\varvec{\epsilon}}}_{1},\boldsymbol{ }{{\varvec{\epsilon}}}_{2},\boldsymbol{ }{{\varvec{\epsilon}}}_{3},{{\varvec{\epsilon}}}_{4}]$ is a vector of residuals. It was assumed that the marker effects can be represented by ${\beta }_{jk}={b}_{jk}\times {d}_{jk}$ where ${b}_{jk}$ is a normal random variable, ${d}_{jk}$ is a dummy (indicator) variable controlling whether the *j*th marker enters in the equation for the *k*th trait, and the ${d}_{jk}$ is assumed to follow a Bernoulli distribution. Thus, both the marker effect and the probability of inclusion may shed light on the importance of a marker on the trait expression.

### GWA for non-additive inheritance models

Initially, all GWA analyses were performed using an additive coding scheme for SNPs (0 = AA, 1 = AB, 2 = BB), assuming a linear change in phenotype based on the number of minor allele copies (allele B). To capture non-additive genetic effects by GWA, we also tested the same MLM and BLINK model after recoding the SNP datasets and filtering for SNPs with all three allelic classes present (*n* = 11,030). In the recessive model, genotypes AA and AB were expected to have similar effects, coded as (0 = AA, AB; 1 = BB). In the dominance model, genotypes AB and BB were treated as having similar effects, coded as (0 = AA; 1 = AB, BB). For the overdominance model, SNPs were coded as (0 = AA and BB; 1 = AB), where both homozygous genotypes were assumed to have the same effect, while the heterozygous genotype exhibited an opposite effect on the trait value (Zhen et al. 2017; Nicolini et al. 2018).

UpSet plots were created to visualize the number of common MTAs between additive and non-additive GWA results using the UpSetR package (Conway et al. 2017). The non-additive MTAs were further assigned to the respective QTLs after inspecting them based on the following rejection criteria: (1) Either of the genotype classes for a SNP represented in fewer than 10 individuals (3% of population); (2) statistical analyses of genotype–phenotype interactions (Tukey’s HSD) did not support the non-additive models; and (3) negligible genetic variance explained (GVE%) by the SNP. The SNP effects were estimated from average BLUE values per genotypic classes: additive effects ($\hat{a}$) by $\widehat{a}= ({\widehat{y}}_{AA}$ —${\widehat{y}}_{BB})/$2 and dominance effects ($\widehat{d}$) by $\widehat{d}={\widehat{y}}_{AB}-({\widehat{y}}_{AA}- {\widehat{y}}_{BB})/2$, where ‘AA’ indicates the group of individuals homozygous for a resistance-associated allele, ‘AB’ heterozygote, and ‘BB’ homozygous for a susceptibility-associated allele of the inspected marker. The degree of dominance was estimated by $|\widehat{d}/\widehat{a}|$ (Walsh 2001; Pincot et al. 2022).

### QTL assignment, pyramiding effect of alleles, and candidate gene search

To assign significant MTAs from the combined across-years phenotypes to distinct QTLs on each chromosome, the physical distance (as base pairs, bp) at which linkage disequilibrium (LD) decayed to the critical value of *r*^2^ = 0.2 was utilized as a threshold to define the boundaries of each QTL region, delineating the range of MTAs encompassed within each QTL. The LD threshold for each chromosome was previously analysed in Rehman et al. (2024) using the 'full-matrix' option in TASSEL version 5.0 (Bradbury et al. 2007).

The contributions of the various QTLs were assessed by fitting multi-locus LMM genetic models for each trait by model (Eq. 7).

7 $${\mathbf{y}}={1\mu} +{\mathbf{M}}{\mathbf{g}}+{\mathbf{Z}}{\mathbf{q}}+{\mathbf{Z}}{\mathbf{u}}+{\mathbf{e}},$$

where **y** is a vector of the phenotypic (BLUE) values of the trait, *μ* is the fixed population mean,** 1** is a vector of ones, and **g**, **q**, **u,** and **e** are vectors of random marker effects, individual non-genetic effects, individual genetic effects, and residual effects, respectively. The **M** matrix has genotypes of the selected *m*_*s*_ markers for the trait. The incidence matrix **Z** relates the observations in **y** to the random-effect vectors **q** and **u**. Assumptions of the random effects were ${\mathbf{g}}_{i} \sim N\left(0,{\sigma }_{M,i}^{2}\right)$ with *i* = 1,…,*m*_s_, $\mathbf{q} \sim N\left(0,\mathbf{I}{\sigma }_{q}^{2}\right)$, $\mathbf{u}\sim N(0,\mathbf{G}{\sigma }_{G}^{2})$ and $\mathbf{e}\sim N(0,\mathbf{I}{\sigma }_{e}^{2})$ where **G** is an ASV-corrected kinship matrix (Feldmann et al. 2022).

Variance components were estimated by REML in sommer::mmer (Covarrubias-Pazaran 2016). The ASV-corrected kinship matrix calculated using AGHmatrix::Gmatrix (Amadeu et al. 2023). The number of QTLs included per LMM model per trait was six for Endpoint, three for AUDPS, two for FPM, and one for GH-LPM. The average semivariance (ASV) method was applied to correct the estimates of the variance components as follows: The ASV-corrected kinship matrix that was applied as the genetic covariance matrix in the LMM (7) resulted in estimates for additive genetic variance (${\widehat{\sigma }}_{G:M}^{2}$) and residual genetic variance $({\widehat{\sigma }}_{{g}_{e}}^{2})$, while the marker-wise variance estimates ${\widehat{\sigma }}_{M}^{2}$ were corrected afterwards by their respective kM bias coefficients (Feldmann et al. 2021, 2022). The final ASV-corrected variance component estimates were then applied to calculate marker-associated phenotypic variance explained, $PVE= {\widehat{\sigma }}_{M}^{2}/({\widehat{\sigma }}_{M }^{2}+{\widehat{\sigma }}_{q }^{2}+{\widehat{\sigma }}_{G }^{2}+ {\widehat{\sigma }}_{e }^{2}/{n}_{r})$, and genetic variance explained, *GVE* = ${\widehat{\sigma }}_{M}^{2}$*∕*${\widehat{\sigma }}_{G}^{2}$, where ${\widehat{\sigma }}_{M}^{2}=\sum_{i=1}^{m}{\widehat{\sigma }}_{M,i}^{2}$. For the equation for PVE, ${n}_{r}$ is the harmonic mean of the number of observations per individual: For the across-year phenotypes from the multi-year field trial, the harmonic means were 4.76 for the leaf aggregate and 3.7 for the FPM datasets, and 7.0 for the leaf PM data from the greenhouse trial.

Probe sequences from previously published powdery mildew resistance QTLs in *F.* × *ananassa* were remapped against the Royal Royce V1 reference genome using the *blastn* tool in Rosaceae.org. The pyramiding effect of favourable alleles was determined based on the most significant marker in each identified QTL, and differences between identified groups were tested by Tukey’s HSD (*p* < 0.05) after testing for homogeneity of variances.

Gene searches were conducted in the ‘Royal Royce’ reference genome version 1 (https://phytozome-next.jgi.doe.gov/info/FxananassaRoyalRoyce_v1_0) within the LD half-decay distance to the top SNP of the inspected QTL. Gene families and most relevant candidate genes were chosen based on the following criteria: (1) genes or orthologs in *F.* × *ananassa* and *F. vesca* with functions associated with PM resistance and (2) homologs in Arabidopsis or other plant species with functions associated with PM resistance.

## Results

### Variation between progenies, years, and phenotyping events

**Fig. 2:**
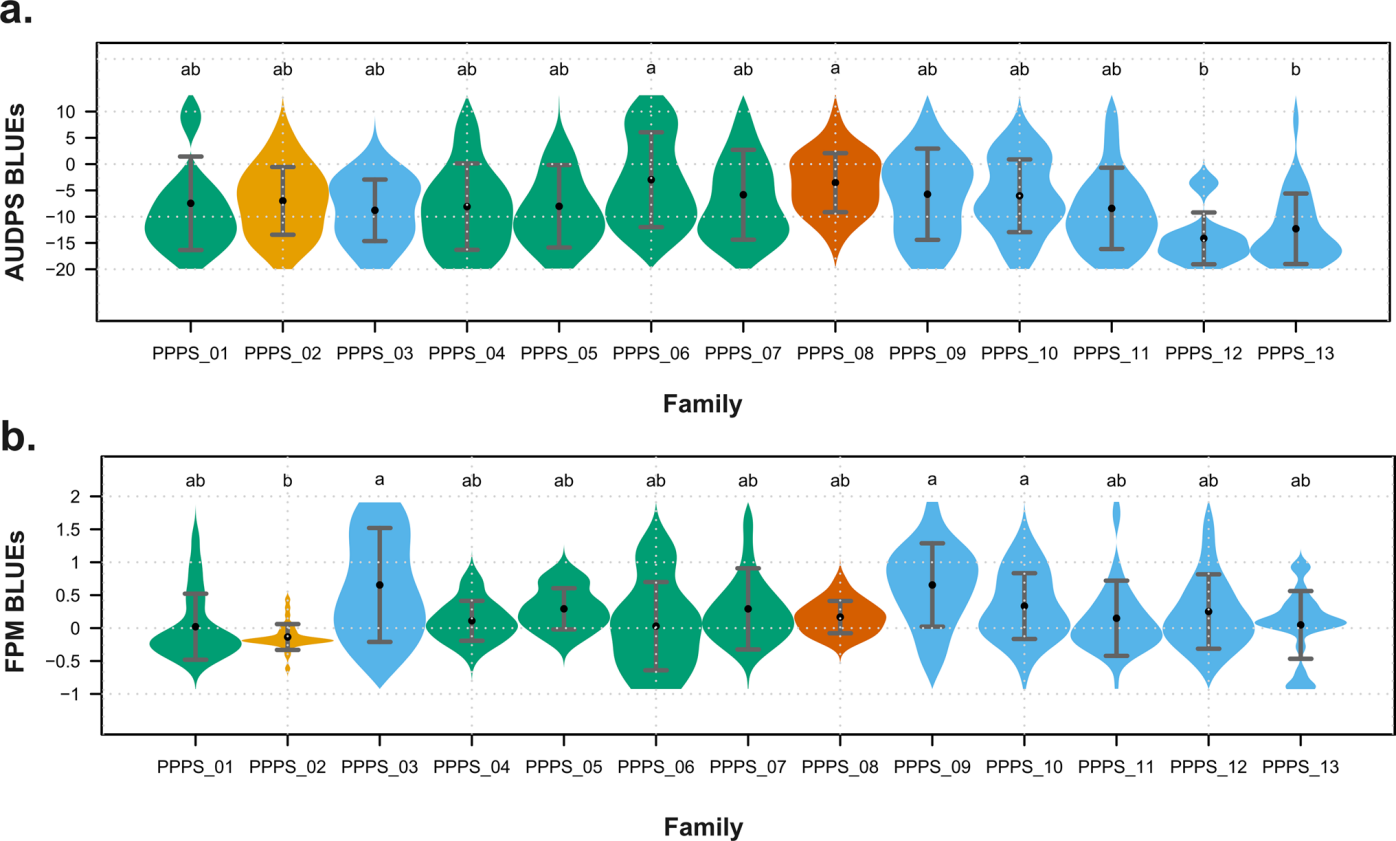
Distribution of leaf powdery mildew AUDPS (**a**) and fruit powdery mildew (**b**) BLUE values in 13 progenies of the ReC population. Grey whiskers represent the standard deviations; black dots show mean values per family. Higher values of BLUEs represent severe disease, and different letters on the top of each violin indicate significant differences based on Tukey’s HSD (*p* < 0.05). Different colours for the violins represent the genetic clustering of progenies

We evaluated clonal plants of 319 ReC strawberry F1 seedlings from 13 full-sib progenies labelled PPPS_01 to PPPS_13 (Table S1) in a replicated field experiment focussing on powdery mildew severity in leaves and fruits (LPM and FPM, respectively) under natural field conditions and tested a subset of the ReC population for LPM severity in a controlled environment. Leaf symptom development in the field trial was less severe in years 2021 and 2022 as compared to 2020 (Fig. S1), with greater variability observed across field plots in 2020 (Fig. S2). Statistically significant differences between the 13 progenies, which were clustered into four groups consistent with pedigree information and sharing of founders (Table S1, Fig. S3) (Rehman et al. 2024), were observed for all PM traits observed in the field experiment (Fig. 2, Fig. S4).

**Fig. 3:**
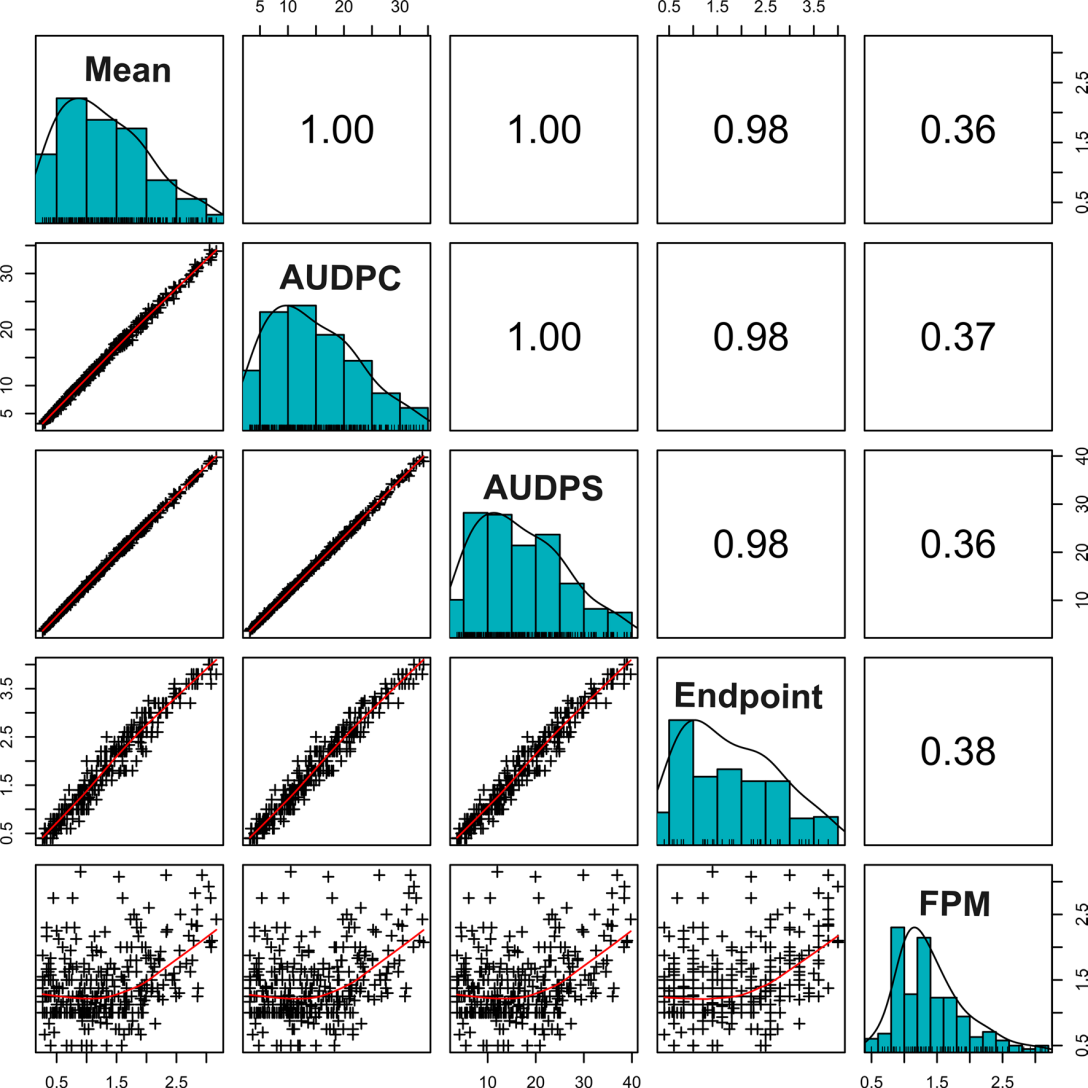
Correlation between different LPM aggregates and FPM for all years combined. The correlation coefficients (*r*) are displayed on the upper diagonal, while scatter plots are shown on the lower diagonal

A highly significant positive correlation ranging from 0.98 to 1 was observed among all LPM aggregates (Fig. 3). Correlation coefficients between years ranged from 0.48 (between 2020 and 2022) to 0.77 (between 2020 and 2021) (Fig. S5). Year-to-year variation was observed in all LPM aggregates, with significant genotype and genotype-by-year interactions (ANOVA: *p* < 0.01) (Table 1, Table S2). A positive correlation was observed between the fruit and leaf powdery mildew aggregates, with coefficients ranging from 0.36 to 0.38 (Fig. 3). Furthermore, the correlation coefficient for FPM between different phenotyping years was 0.45 (Fig. S5).

**Table 1:** Descriptive statistics, variance components, and broad-sense heritabilities for leaf and fruit powdery mildew disease aggregates of strawberry ReC population in the field trial

Year	Tissue	Aggregate	Mean	SD^a^	CV^b^	var_G_^c^	var_E_^d^	H2^e^
2020	Leaf	Endpoint	2.61	1.10	0.42	0.89	0.25	0.87
		Mean	1.91	0.77	0.40	0.44	0.12	0.87
		AUDPC	22.35	9.07	0.40	60.45	16.94	0.87
		AUDPS	25.82	10.48	0.40	80.97	22.31	0.87
2021	Leaf	Endpoint	1.27	1.22	0.95	1.18	0.23	0.90
		Mean	0.97	0.95	0.97	0.74	0.12	0.92
		AUDPC	10.46	10.14	0.96	83.45	14.99	0.91
		AUDPS	12.10	11.74	0.97	112.66	19.38	0.91
	Fruit	FPM	1.02	0.68	0.44	0.39	0.10	0.86
2022	Leaf	Endpoint	1.01	1.11	1.08	0.78	0.34	0.67
		Mean	0.64	0.80	1.25	0.49	0.09	0.81
		AUDPC	5.83	7.66	1.31	45.02	8.11	0.82
		AUDPS	7.26	9.20	1.26	62.29	12.14	0.81
	Fruit	FPM	0.63	0.52	0.44	0.14	0.13	0.63
All years	Leaf	Endpoint	1.78	1.35	0.75	0.82	0.44	0.64
		Mean	1.30	1.00	0.77	0.47	0.23	0.69
		AUDPC	14.53	11.48	0.79	55.13	27.70	0.66
		AUDPS	16.88	13.24	0.78	75.24	36.86	0.67
	Fruit	FPM	1.44	0.68	0.47	0.19	0.20	0.48

^a^SD is standard deviation calculated as the square root of sample variance

^b^CV refers to the coefficient of variation, computed as the ratio of the SD to the mean

^c,d^var_G_ and var_E_ refer to the genotypic and environmental variance, respectively

^e^H2 refers to the broad-sense heritability for the respective trait

The broad-sense heritability estimates for resistance to powdery mildew in leaves were higher for the initial two years compared to the third year of the field experiment, with overall heritabilities ranging between 0.64 and 0.69 for different LPM aggregates. According to correlation analyses, we found the disease aggregates Endpoint and AUDPS most informative; consequently, they were selected as the representative phenotypes for subsequent analysis. For fruit powdery mildew, broad-sense heritabilities for the two consecutive years ranged from 0.63 to 0.86, with overall value of 0.48. These results indicated that a large proportion of the variation in both traits was genetically controlled (Table 1).

**Fig. 4:**
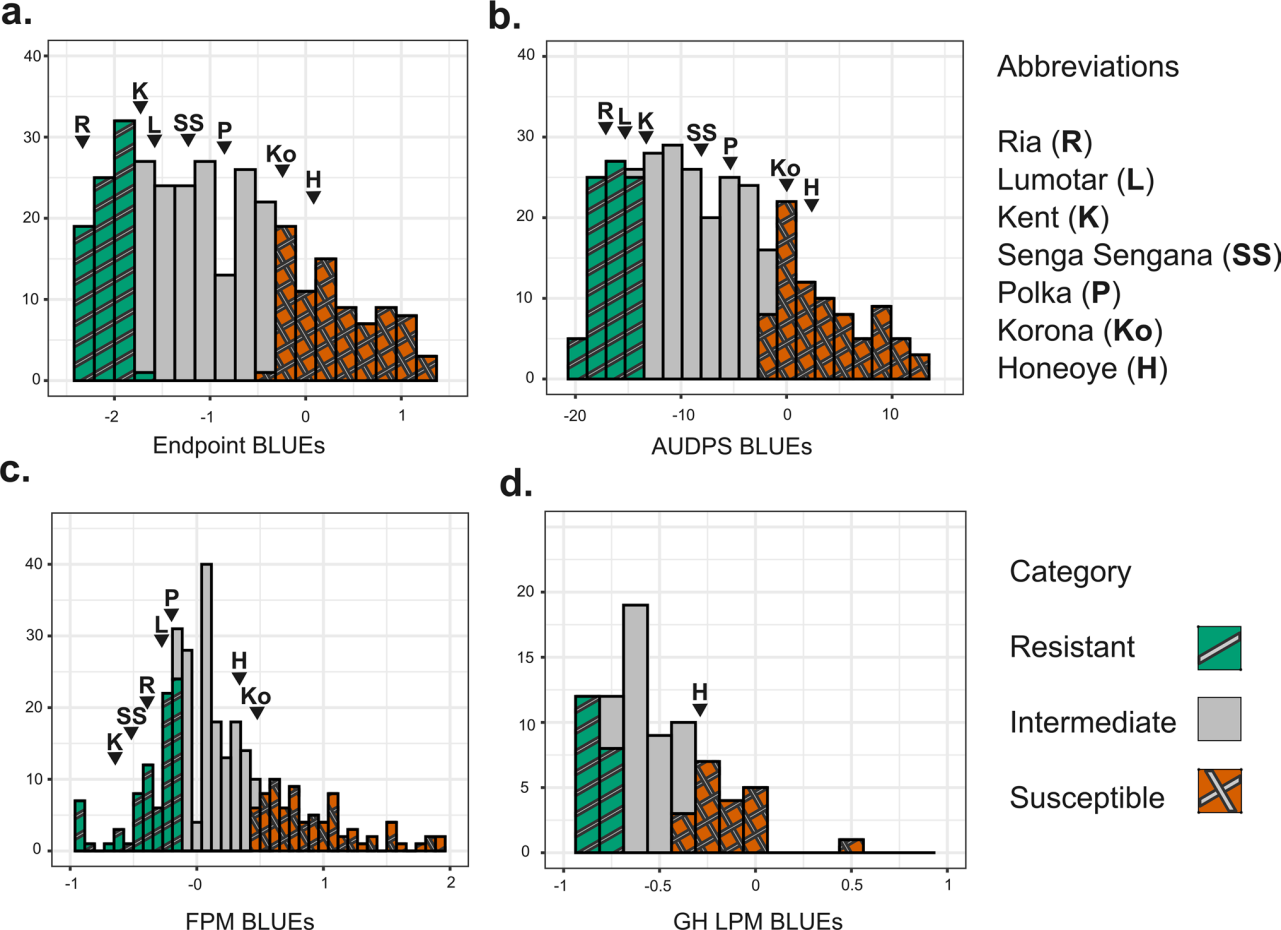
Frequency distribution of BLUEs of leaf powdery mildew aggregates in the field trial (**a**, **b**), fruit powdery mildew in the field trial (**c**), and leaf powdery mildew in the greenhouse trial (**d**). Different categories are coloured based on the top and bottom 30% of the respective BLUE values

A large proportion of the ReC individuals showed resistance to LPM, with approximately 30% of the population exhibiting no or negligible symptoms across years; this segment was classified as resistant. Similarly, a comparable portion of the ReC population showed severe disease symptoms and hence classified as susceptible. Among the cultivars used as controls, ‘Honeoye’ and ‘Korona’ were susceptible, ‘Polka’ intermediate, whereas ‘Lumotar’, ‘Kent’, ‘Senga Sengana’, and ‘Ria’ showed only mild symptoms (Fig. 4).

### Number of MTAs varied between years, GWA models, and LPM aggregates

Various GWA models were tested, and while FarmCPU did not offer additional benefits over MLM and BLINK, the latter models were chosen, as their QQ plots demonstrated effective control of inflation risk and identified most of the MTAs of interest for all the traits (Fig. S6). A total number of 76 MTAs for LPM aggregates and 14 MTAs for FPM were observed in our GWA with both the chosen models. The number of MTAs varied between different phenotyping years, GWA models, and LPM aggregates (Fig. S7a, b, c). Among the tested models, the MLM model identified the largest number of MTAs, while the BLINK model identified the least, yet the most unique ones. However, all models shared a considerable number of MTAs, signifying the consistency of the GWA analyses. The combined analysis of data spanning three seasons for the two LPM aggregates (Endpoint and AUDPS) revealed variation in the total number of MTAs observed among these aggregates based on respective -log(*p*) values (Figs. S7, S8, S9). For FPM, we found 13 MTAs in common with the LPM from the field experiment but none with the greenhouse validation experiment (Figs. S7, S10). The common and unique loci identified for all the PM traits across models and years are visualized as a summary plot (Fig. S7) with detailed information provided in Dataset S2 and the year-wise Manhattan plots in Figs. S8 and S9.

### Identification of stable PM Resistance QTLs with additive GWA

QTLs that were stable for PM resistance across years were then identified by the additive GWA MTAs of combined-year BLUEs for LPM (Endpoint and AUDPS) and FPM from the field trial. Respective MTAs amounted to eight QTLs distributed across six chromosomes: six for LPM and two for FPM (Fig. S7, Tables 2, 3). Detailed information on all MTAs associated with each QTL per year, per model, and per disease aggregate is provided in Dataset S2. Notably, two overlapping QTLs (q.LPM.Rec-5B and q.LPM.Rec-7D) have previously been reported for LPM in garden strawberry, while no FPM QTLs have been described in the literature (Table 2). The identified QTLs for both leaf and fruit PM explained considerable phenotypic and genetic variance, as indicated by PVE and GVE values calculated using ASV-corrected REML estimates (Table 3).

**Table 2:** Leaf powdery mildew QTLs and their top SNPs ID, physical position (bp), *p* value, minor allele frequency (MAF), and estimates of additive (â) and dominance (^d) effects and dominance degree (^d/â) for the respective GWA approach per the most significant SNP

QTL	Top SNPs	Chr	Position	*p* Value	MAF	GWA model	GWA approach	â	^d	|d/a|
*Leaf*										
q.LPM.Rec-3B.1 *	*AX.184227292*	3B	3 705 268	2.39E−09	0.43	BLINK	Additive	− 3.41	− 0.87	0.3
				2.80E−06	0.08	BLINK	Recessive			
q.LPM.Rec-3B.2 *	*AX.184098865*	3B	29 912 198	1.47E−15	0.21	MLM	Additive			
				3.73E−09	0.21	BLINK	Additive			
	*AX.184571439*	3B	28 214 103	1.37E−08	0.26	MLM	Dominant	− 2.74	4.66	1.7
	*AX.184895875*	3B	29 362 633	1.57E−08	0.40	MLM	Additive	− 5.54	− 3.25	0.6
				1.80E−06	0.08	MLM	Recessive			
				2.11E−08	0.08	BLINK	Recessive			
	*AX.184751456*	3B	29 787 819	2.49E−11	0.24	MLM	Recessive	− 4.58	3.14	0.7
*q.LPM.Rec-5B †*	*AX.184199976*	5B	8 817 140	1.45E−06	0.25	BLINK	Additive			
q.LPM.Rec-6A *	*AX.184960774*	6A	34 273 789	1.54E−06	0.36	BLINK	Additive	− 0.90	− 3.58	4.0
				1.23E−08	0.25	BLINK	Recessive			
*q.LPM.Rec-6B †*	*AX.184633416*	6B	2 561 272	2.16E−06	0.22	BLINK	Additive			
*q.LPM.Rec-7D †3*	*AX.184357125*	7D	19 668 633	1.80E−06	0.43	BLINK	Additive	− 3.18	− 2.20	0.7
	*AX.184037708*	7D	19 287 746	2.11E−07	0.07	BLINK	Recessive	− 2.55	− 1.17	0.5
*Fruit*										
*q.FPM.Rec-3B*	*AX.184327796*	3B	30 278 425	1.18E−06	0.24	MLM	Additive	− 0.21	− 0.58	2.8
*q.FPM.Rec-4B*	*AX.184557621*	4B	10 404 300	1.17E−06	0.46	BLINK	Additive	− 0.57	− 0.17	0.3
*Greenhouse leaf*										
*q.LPM_GH.Rec-3B*	*AX.184652123*	3B	27 935 377	1.44E−11	0.26	BLINK	Additive			

The â, ^d, and ^d/â values are only calculated for SNPs with all three allelic classes present in the population. In the case of leaf PM, the additive, dominance, and dominance degree are shown for the Endpoint aggregate

†Endpoint only *Both Endpoint and AUDPS

**Table 3:** Tissue-specific powdery mildew resistance QTLs, their focal SNP, marker-associated phenotypic (PVE) and genetic (GVE) variances explained

Trait	QTL	Chr	Focal SNP	Across years		2020		2021		2022		Colocalized QTLs from previous publications^a^ and from this study*
				PVE (%)	GVE (%)	PVE (%)	GVE (%)	PVE (%)	GVE (%)	PVE (%)	GVE (%)	
Leaf Endpoint	*q.LPM.Rec-3B.2*	3B	AX.184895875	41.6	45.1	28.6	37.0	42.4	51.9	12.2	21.4	
	*q.LPM.Rec-3B.1*	3B	AX.184227292	6.2	6.7	7.5	9.6	2.3	2.8	4.2	7.4	
	*q.LPM.Rec-7D*	7D	AX.184037708	8.8	9.6	1.1	1.4	9.0	11.0	13.3	23.4	FaRPa7D1 (0.7 Mb)^1^
	*q.LPM.Rec-5B*	5B	AX.184199976	4.3	4.7	2.7	3.5	3.1	3.8	4.5	7.9	FaRPa5B (0.4 Mb)^1^
	*q.LPM.Rec-6A*	6A	AX.184960774	2.7	3.0	2.3	3.0	2.1	2.6	2.8	4.9	
	*q.LPM.Rec-6B*	6B	AX.184633416	2.5	2.7	1.7	2.1	3.0	3.7	0.1	0.2	
Leaf AUDPS	*q.LPM.Rec-3B.2*	3B	AX.184895875	40.8	45.4	22.1	30.2	42.0	53.4	14.6	29.8	
	*q.LPM.Rec-3B.1*	3B	AX.184227292	7.4	8.2	9.1	12.4	2.5	3.2	5.8	11.9	
	*q.LPM.Rec-6A*	6A	AX.184960774	3.3	3.7	2.5	3.4	2.6	3.3	2.4	4.8	
Fruit	*q.FPM.Rec-3B*	3B	AX.184327796	17.1	22.7			12.6	21.5	20.4	39.0	q.LPM.Rec-3B.2^*^
	*q.FPM.Rec-4B*	4B	AX.184557621	27.6	36.6			23.9	40.8	8.1	15.4	
Greenhouse leaf	*q.LPM.GH.Rec-3B*	3B	AX.184652123	31.3	32.2							q.LPM.Rec-3B.2^*^

The major QTL on chromosome 3B and its colocalized QTLs are underlined

^1^Cockerton et al. (2018) for leaf powdery mildew resistance in field conditions

While the MLM model revealed several significant MTAs, the application of BLINK algorithms proved to be more powerful as expected, and many unique QTLs were identified exclusively by this approach (Huang et al. 2019) (Tables 2, 3). The MLM model for LPM revealed only one on chromosome 3B while BLINK models revealed five QTLs on five different chromosomes in addition to the one on 3B (Table 2). However, for FPM, the MLM and BLINK model detected one QTL region each on two chromosomes, q.FPM.Rec-3B and q.FPM.Rec-4B, respectively (Table 3).

### LPM QTLs identified by non-additive effect GWA

**Fig. 5:**
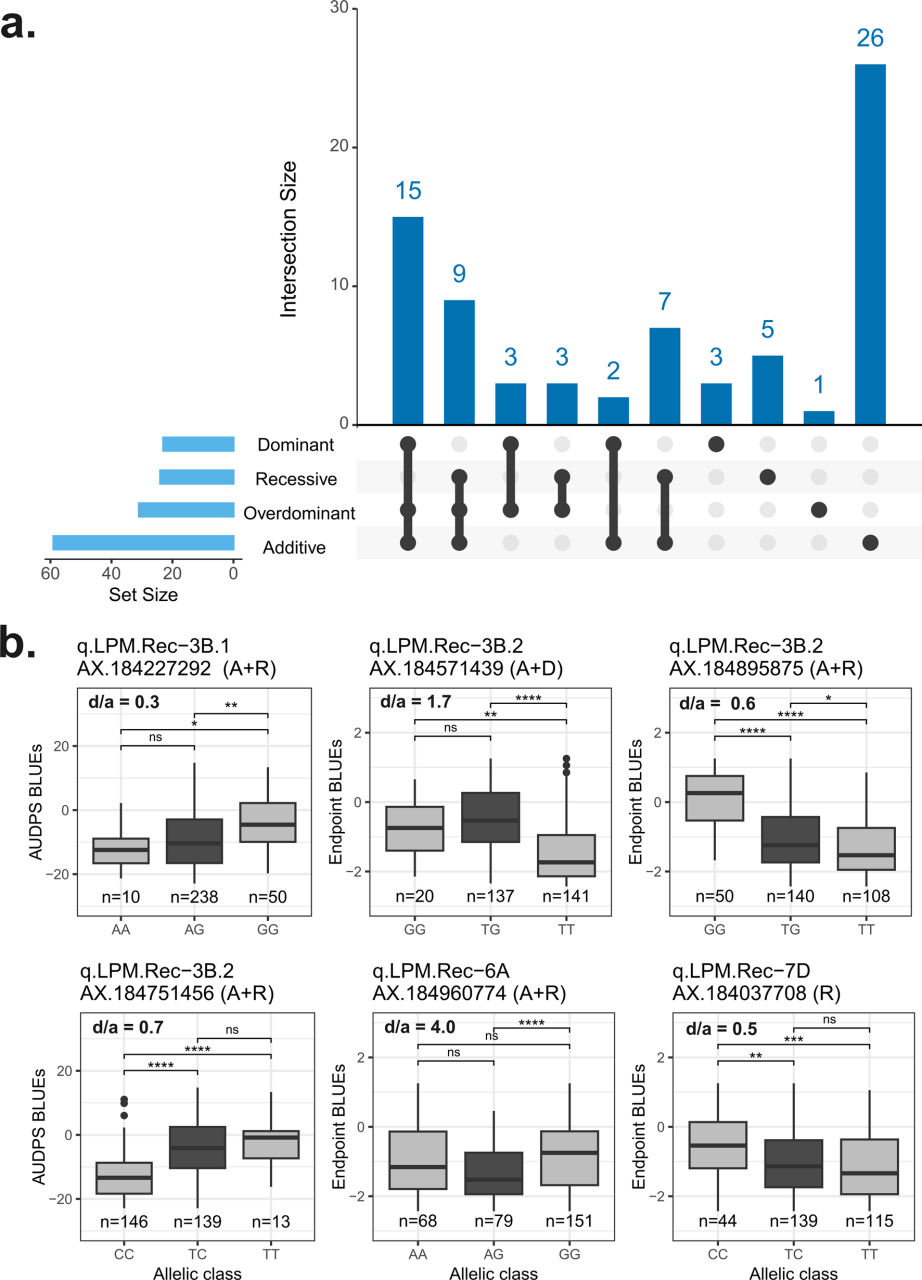
Non-additive GWA for leaf powdery mildew, with overlapping sets of associated SNPs between additive and non-additive models (**a**) and candidate SNPs for non-additive allelic effects within four QTLs (**b**). The UpSet plot in **a** highlights the interactions between different GWA approaches: The bar chart on the left indicates the total count of MTAs identified by each allelic coding, while the upper bar chart displays the intersection sizes between their counts. The dark dots and connected lines in the bottom panel indicate which models contribute to each intersection, with single dots representing unique models. The allelic boxplots in **b** have the respective significant coding model(s) after the SNP probe name in parentheses: A, additive and R, recessive. The values for dominance degree (*d*/*a*) for each SNP are shown inside the plot. Asterisks on the box plots denote significant differences between allelic classes, with significance levels from post-GWA Tukey’s HSD test are marked as: **** (*p* < 0.0001), *** (*p* < 0.001), ** (*p* < 0.01), * (*p* < 0.05), and ns (*p* > *0.05*). Allelic classes are shown as homozygous (white) and heterozygous (grey)

Using both MLM and BLINK GWA models on combined across-years LPM traits, we analysed recessive, dominant, and overdominant associations, identifying a total of 74 MTAs that passed the stringent Bonferroni threshold (− log(*p*) = 5.61). Our GWA analysis revealed both unique and shared MTAs across the various non-additive models and the additive model, distributed across eight chromosomes (Fig. 5a, Dataset S2). Among these significant MTAs and the resulting QTLs, six were confirmed as true associations after inspecting the phenotype–genotype associations, while others were not well supported by phenotype distribution and GVE criteria (Fig. 5b). The final non-additive QTLs, which were identified by using recessive or dominant coding for six, and overdominant coding for one, of the LPM QTLs, overlapped with the additive QTLs and also sometimes shared the same top SNP (Table 2). When inspecting the phenotype associations within the non-additive QTLs, those on chromosomes 3B (3B.1 and 3B.2) and 7D demonstrated strong candidate SNPs that exhibited predominantly either recessive or dominant expression of the trait and were supported by degree-of-dominance values close to 1 (Fig. 5b, Table 2). However, the candidate SNP for the overdominance coding on chromosome 3B was not statistically supported and appeared recessive, while the one for the recessive coding on 6A was not fully statistically supported (Fig. 5b). Overall, our results suggest the presence of both additive and non-additive genetic effects for LPM in the ReC strawberry population, with the overlap between the models indicating partial dominance, allelic heterogeneity, or locus heterogeneity within these QTLs.

### Fruit PM associated with one unique and one LPM QTL

In the ReC population, we identified two FPM QTLs, including one unique for fruit-specific PM response and one overlapping with the leaf resistance QTL, q.LPM.Rec-3B.2, located on chromosome 3B (Table 3). The presence of a genetic factor unique to FPM or to LPM resistance is in accordance with separate genetic factors responsible for PM resistance in different tissues of garden strawberry (Tapia et al. 2021a, b; Lynn et al. 2024). The fruit-specific QTL on chromosome 4B explained 37% of the GVE and to our knowledge is novel (Table 3).

### Validation of LPM and FPM QTL on 3B by multiple-trait modelling

We then applied Bayesian multivariate modelling for FPM and LPM phenotypes from the field trial data from two years. The SNPs within the major QTL on 3B showed elevated effects both on LPM and FPM traits in both years (Figs. S11, S12, Dataset S2). The effect sizes and probabilities of inclusion for the SNPs of the 3B QTL were greater for the modelled traits of the year 2021 than those of the year 2022, consistent with the heritability estimates of the same traits and the same years (Table 1). Overall and for the major QTL region on 3B, q.LPM.Rec-3B.2 (Figs. S13, S14), the SNPs effects were same-directional and correlated, indicating that SNPs with large effects on LPM had large effects on FPM, too.

### Validation of LPM GWA in controlled conditions

To further test the stability of our GWA results, we conducted an independent replicated experiment in a controlled greenhouse environment using a subset of 78 ReC individuals. These individuals were evaluated specifically for LPM resistance (Dataset S1). In this validation study, we identified one QTL present on chromosomes 3B coinciding with the respective LPM QTLs identified in the initial field experiment for LPM (Fig. S10, Table 2), highlighting the robustness of our findings. The detection of the same genetic region under different environmental conditions underscores the reliability of the identified QTL and its potential application in breeding programmes aimed to enhance PM resistance in garden strawberry.

### A stable major QTL associated with PM resistance and candidate gene analysis

**Fig. 6:**
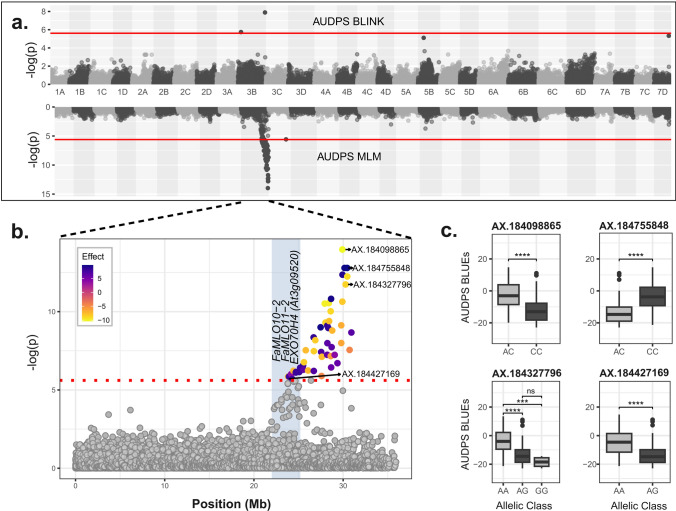
Chromosomal locations of SNP markers associated with leaf powdery mildew resistance (**a**, **b**) and allelic effects of selected SNPs detected within a major QTL in 3B (**c**). The significant MTAs are shown as a Miami plot for AUDPS using BLINK (upper) and MLM (lower) models (**a**), a chromosome 3B Manhattan plot highlighting significant MTAs for q.LPM.Rec-3B.2 by MLM (b), and as phenotype–genotype association box plots for its selected SNPs (**c**). Red lines in both **a** and **b** indicate the FDR threshold at − log10(*p*) = 5.61. SNPs passing the Bonferroni threshold and exhibiting the highest GVE (%) values are highlighted in **a** and coloured in **b** based on the respective SNP effects from the MLM model. Names of the major candidate genes are indicated by their relative physical positions in the blue shaded area in panel **b**. SNP effect box plots for the highlighted SNPs have their respective GVE (%) in parenthesis while the asterisks on box plots indicate significant differences between different allelic classes (Tukey’s HSD; *p* < 0.0001 = ****, *p* < 0.01 = ***, and *p* > 0.05 = ns) (**c**)

Across all three phenotyping years in the field and the greenhouse validation experiment, GWA models and GWA approaches (both additive and non-additive), the QTL q.LPM.Rec-3B.2 that confers LPM resistance was consistently detected at ≈ 22–30 Mb on chromosome 3B. It had varying numbers of MTAs identified by both MLM and BLINK models for each independent year, i.e. 2020 (*n* = 44), 2021 (*n* = 68), 2022 (*n* = 0), and for all the years combined (*n* = 60) (Dataset S2). Importantly, the top SNP (AX-184098865) remained the same for the combined analysis, as well as for the years 2020 and 2021. Although no significant GWA signals surpassed the Bonferroni threshold in 2022, an emerging signal on chromosome 3B, similar to those observed in 2020, 2021, and the combined analysis, was observed, with the top SNP approaching significance (Figs. S8, S9). The same top SNP for the LPM QTL q.LPM.Rec-3B.2 remained consistent across both MLM and BLINK. We also inspected three additional SNPs from the additive MLM model in the same region (Fig. 6). For the QTL on 3B.2, we selected AX.184895875 as the focal SNP according to its effect size and allelic balance. This consistent QTL on chromosome 3B explained on average 45% of the genetic variance associated with leaf resistance in the field, 32% in the greenhouse, and 23% of fruit resistance, remaining high across all field trial years (Table 3).

Subsequently, we searched candidate genes within the stable major QTL region on 3B that we identified for powdery mildew resistance in both strawberry leaves and fruits. Gene searches were performed in the strawberry genome model within, and proximate to, the QTLs. A comprehensive list of these genes is presented in Dataset S3. We narrowed down the pool of potential candidate genes through an examination of the functional information, proximity, and previously reported roles of the same gene families, or of other homologous or homoeologous genes, in PM response (Table 4).

**Table 4:** List of candidate genes of interest, along with their positional information, identified in the region harbouring the novel major QTL associated with powdery mildew response in reconstructed strawberry

QTL	Candidate gene	Literature source	Homologous gene ID	Start	End
q.LPM.Rec-3B.2				21 734 451	30 924 079
	FaMLO10-2	Tapia et al. (2021b)	Fxa3Bg202817.1	22 900 579	22 906 007
	FaMLO11-2	Tapia et al. (2021b)	Fxa3Bg202818.1	22 942 041	22 948 411
	EXO70H4 (At3g09520)	Huebbers et al. (2024)	Fxa3Bg202957.1	24 160 718	24 162 487
	PMR5	Vogel et al. (2004)	Fxa3Bg203302.1	27 015 763	27 018 829
	LRR	Wang et al. (2022)	Fxa3Bg203855.1	30 885 186	30 890 078

### The pyramiding effect of favourable alleles

**Fig. 7:**
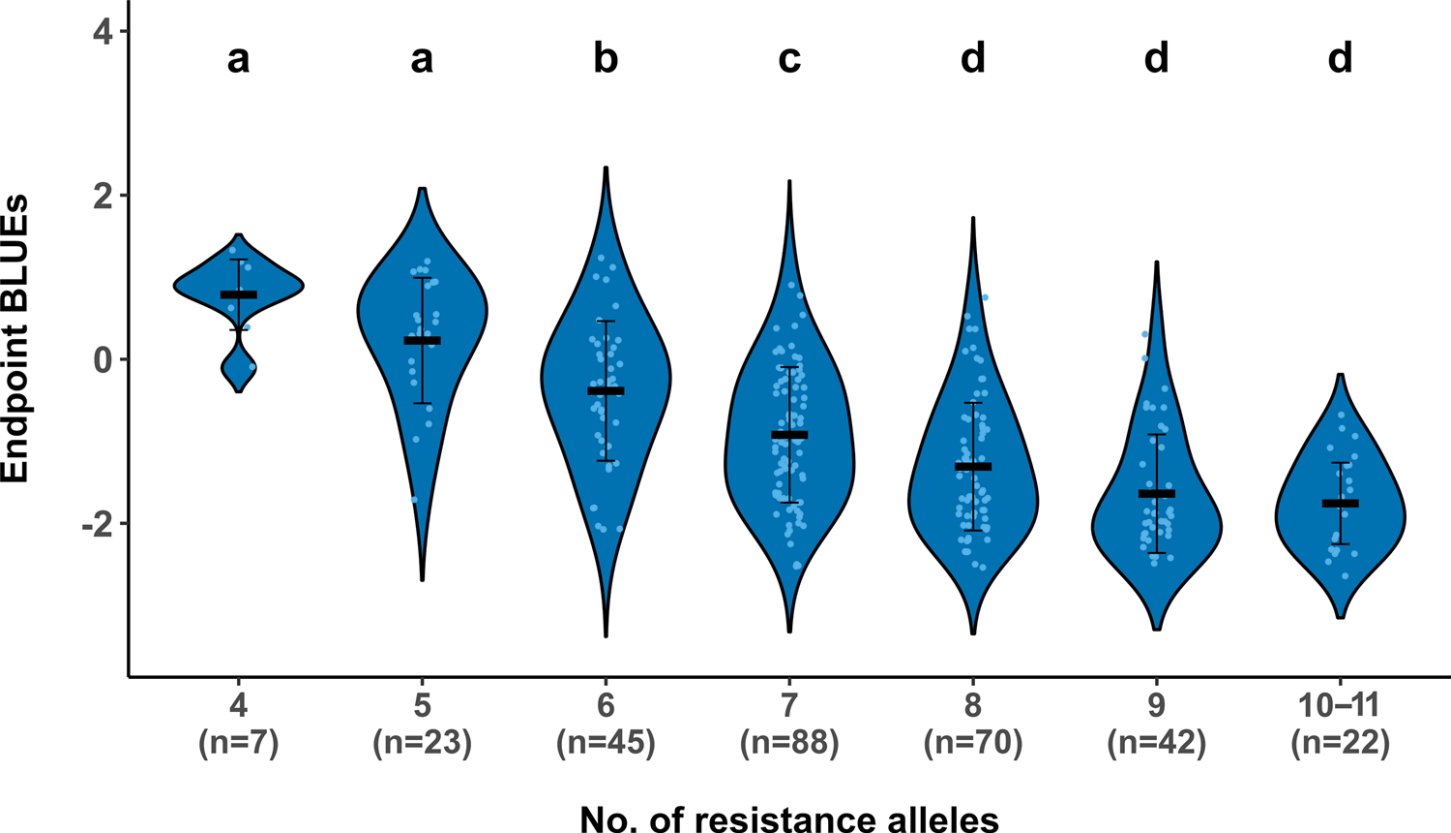
The cumulative effect of resistance alleles of the six leaf powdery mildew QTLs on leaf Endpoint BLUEs in the ReC strawberry population. The number of individuals in each genotype group is mentioned in parentheses. Black bars and whiskers represent the mean and standard deviation per group, respectively. Different letters on the violins indicate significant differences between the groups based on Tukey’s HSD (*p* < 0.05)

PM resistance is a complex trait controlled by multiple QTLs conferring resistance or susceptibility to the fungus. We tested the cumulative effect of favourable alleles that were associated with resistance to LPM by assigning ReC individuals to groups based on the number of resistance-associated alleles carried in each genotype. Across the six QTLs detected by additive GWA in the ReC population for LPM resistance across years, at most 11 resistance-associated alleles found in any one accession. ReC individual groups that possessed six or more resistance-associated alleles were significantly more resistant to the groups with a lower number (four to five) of such alleles (Fig. 7).

## Discussion

Understanding genetic factors involved in polygenic resistance against powdery mildew in garden strawberry is very helpful for enhancing its resistance breeding. Here, we employed multi-model GWA to identify QTL regions associated with PM resistance for both leaves and fruits using a diverse pre-breeding strawberry population. Our investigation revealed numerous QTLs with varying effects linked to LPM and FPM disease resistance, including a new major QTL on chromosome 3B associated with both traits. Our results provide information on resistant pre-breeding germplasm resources that have the potential to offer novel variation for future genome-informed breeding efforts in garden strawberry.

### PM resistance exhibited strong genetic control in strawberry leaves and fruits

The phenotypic data presented in this study displayed a nearly continuous distribution, in accordance with the quantitative nature of PM resistance. The ReC population was predominantly resistant or partially resistant, though it also contained susceptible and highly susceptible individuals. Importantly, in comparison with the climatically adapted control cultivars used as standards, the best-performing portion of ReC population were more resistant than the best cultivars (Ria, Kent, and Lumotar). LPM resistance in the ReC population expressed strong genetic control, as shown by broad-sense heritability values exceeding 80% in all three years. These H^2^ values are consistent with the findings of Lynn et al. (2024) and Nelson et al. (1995) but are higher than those reported by Tapia et al. (2021a) and Davik and Honne (2005), who found moderate heritability levels for LPM in garden strawberry. Heritability values are usually influenced by the variation present in the tested plant material, indicating that our ReC population possesses a considerable amount of genetically controlled variation for all LPM aggregates. The observed variation in ReC population is useful for breeding purposes, as selected individuals could be a valuable source of resistance in garden strawberry breeding programmes.

Strong correlations were observed between LPM scores from the years 2020, 2021, and 2022, further supporting that a robust genetic component governs PM resistance in leaves. Variations in disease resistance across the three phenotyping years may be attributed to differences in disease pressure or environmental factors, such as the weather (Blanco et al. 2004; Carisse et al. 2013). Similar heritability estimates (> 80%) were found for FPM in 2021; however, the heritability for FPM in 2022 was moderate, with genetic factors accounting for 63% of the observed variation, slightly higher than the score of 53% reported by Lynn et al. (2024).

Very high correlations were found between different LPM aggregates. Noteworthy, while AUDPC and AUDPS assess similar aspects of disease progression, they differ in the weight that is given to the first and last observations. AUDPC applies the ‘trapezoidal method’, which gives less weight to the first and last observations, potentially underestimating disease progression when time intervals between observations are unequal. In contrast, AUDPS employs the ‘staircase method’, which assigns equal weight to all observations, including the first and last, offering a more consistent representation of disease progression across irregular time intervals (Simko and Piepho 2012). Hence, we determined AUDPS to be a particularly well-suited aggregate for LPM assessment and consequently used it in association mapping, together with Endpoint.

### Multi-model GWA revealed the QTL landscape for PM resistance in strawberry

We conducted GWA using year-specific PM observations and compared year-specific MTAs to MTAs from the combined phenotypes across years (Dataset S2). We report six QTL regions based on high confidence BLUE phenotypes for field LPM across years. In comparison with previously published markers mapped from progenies of garden strawberry (Cockerton et al. 2018; Sargent et al. 2019; Lynn et al. 2024), potential overlap was found in two QTLs identified in our ReC population on the chromosomes 5B and 7D (Cockerton et al. 2018). Hence, these QTL regions may be relevant for PM resistance across environments and across the cultivated and the wild-derived strawberry germplasms. The rest of our ReC QTLs may represent novel resistance factors.

The single-locus-based MLM involves a one-dimensional genome scan, iteratively testing one marker at a time, which may not be the best-performing model for dissecting complex traits such as strawberry PM resistance. Hence, we also employed two multi-locus GWA approaches, FarmCPU and BLINK, which have previously been successful in dissecting several horticultural traits in the same ReC population (Rehman et al. 2024). FarmCPU addresses confounding factors by testing associated markers as covariates through a fixed-effects model to control for false positives. It further optimizes associated covariate markers using a random-effect model (Liu et al. 2016). Conversely, BLINK enhances bin size optimization by considering marker linkage, thereby improving computational speed and statistical power (Huang et al. 2019). While multi-locus GWA models are generally considered superior to single-locus GWA models, no single model suits all situations, especially for traits with a pleiotropic nature (Tibbs Cortes et al. 2021). Therefore, testing different models to dissect the unique genetic architecture of complex traits is recommended. In our current study, FarmCPU and BLINK produced fairly similar results, with both identifying multiple QTLs that were not detected by the single-locus MLM model. Given this similarity, we focus on presenting the results from BLINK, which demonstrated multiple unique QTLs that were not identified by MLM. Such numerous small-effect QTLs likely contribute to the predicted total genetic values for complex traits (Meuwissen et al. 2001) and, apart from marker-assisted selection, could be useful in genomic selection for PM resistance. Indeed, we found that multiple QTLs contribute quantitatively to increasing resistance (Fig. 7). Development of genomic selection for PM resistance, such as done by Tapia et al. (2022) for *F.* × *ananassa* breeding populations, could provide additional benefits over marker-assisted selection and could be inspected in future.

### Non-additive GWA suggested mode of inheritance for several of the QTLs

Traditionally, GWA has focussed solely on the additive effects of individual SNPs (Bush and Moore 2012; Marjoram et al. 2014). Nevertheless, we have demonstrated the additional benefit of incorporating non-additive effects by identifying five QTLs that include SNPs having a non-additive phenotype distribution between genotypes. This indicates that a considerable proportion of the genetic variation in our hybrid population may be attributable to non-additive effects (Fig. 5). The previous genetic studies have highlighted the presence of both additive and non-additive variance in powdery mildew resistance. Both Simpson (1987) and Davik and Honne (2005) claimed the additive genetic variance to be the most important part while others report that the non-additive part dominates (e.g. Daubeny 1961; Hsu et al 1969; Masny et al 2016). Cockerton et al (2018) and Lynn et al (2024) used DNA-based information to identify the types of genetic effects; they report additive ones for their associated SNPs. Our multi-model GWA approach demonstrated that alleles underlying LPM variation can be identified when non-additive effects are explicitly considered in a GWA setting, thereby enhancing the ability to dissect complex traits in crop plants.

Due to the underlying assumption of additive effects in traditional GWA, it is likely that additive ones are overrepresented in the GWA literature. However, recessive and dominant effects are highly relevant for many resistances, including resistance to PM in different plants (Pryor 1987; Adam and Somerville 1996; Azmat et al. 2010; Piechota et al. 2020). Our study leverages genomic data to report various QTLs with putative dominant modes of resistance to powdery mildew. While the previous studies in strawberry and other crops have identified, through controlled crosses, QTLs for various traits with recessive or dominant effects (Wallace et al. 2014; Cui et al. 2017; Pincot et al 2018), our research also highlights the relevance of non-additive models alongside additive GWA which has identified most QTLs. The importance of non-additive GWA is increasingly recognized in crop plants, and tools such as ‘GWASpoly’ (Rosyara et al. 2016) enable automated testing of dominance models for both diploid and polyploid species, reducing the need for manually recode SNP datasets. Moreover, the concept of overdominance, where the heterozygous genotype confers greater resistance than either homozygous genotype, also warrants attention, particularly when dealing with clonal or hybrid breeding systems.

### A stable QTL on 3B and its candidate genes

In addition to several small-effect QTLs, one major QTL region—q.LPM.Rec-3B.2 conferring LPM resistance—was consistently detected between approximately positions 22 and 30 Mb on chromosome 3B. This QTL was identified across all phenotyping events and aggregates for LPM, as well as in the greenhouse validation subset. The QTL explained a large portion of the phenotypic and genetic variation of leaf disease symptoms across the years and the two environments, making it both a major and an environmentally stable QTL region for powdery mildew resistance. The detection of the same region for FPM identified both by single-trait GWA and by multivariate analyses, further emphasizes the importance of this QTL, which possibly harbours candidate genes for general PM resistance in strawberry.

It has been suggested that different genes may confer resistance to PM depending on the inoculum level (Nelson 1995, 1996; Kennedy et al. 2013). However, the *Mildew Resistance Locus O* (*MLO*) gene family is a key target for enhancing resistance against PM pathogens across various crops. *MLO* genes are known to be involved in PM susceptibility, and their loss-of-function or silencing can confer durable and broad-spectrum resistance against the pathogen (Kusch and Panstruga 2017; Tapia et al. 2021b). Transgenic downregulation or elimination of specific endogenous *MLO* gene sequences has led to resistance against PM, with the first report of *MLO*-based PM resistance coming from studies in barley (Jørgensen 1992). We identified two *MLO* genes near our stable QTL (q.LPM.Rec-3B.2) on chromosome 3B, including *FaMLO10*, which belongs to the functionally characterized clade V *MLO* genes commonly linked to PM susceptibility in dicots (Kusch and Panstruga 2017) and whose transient silencing reduces PM susceptibility in garden strawberry leaves (Tapia et al. 2021a, b). In addition, we found the EXO70 subunit family gene (*EXO70H4*) close to the major ReC QTL on chromosome 3B. A recent study has shown that double mutants lacking the expression of EXO70H4 and either of the clade V MLO family proteins MLO2 or MLO6 have reduced susceptibility to powdery mildew in Arabidopsis (Huebbers et al. 2024). MLO proteins play a role in exocytosis, the targeted delivery of proteins to the extracellular space, which is crucial for cell wall construction. The EXO70 family of proteins, as part of the exocyst complex, aids in the docking of vesicles carrying these materials to the plasma membrane. Thus, the pleiotropic effects of these two families in Arabidopsis PM response could be explained by their synergistic role in cell wall formation.

Additional candidates for resistance factors near the major QTL on chromosome 3B include genes containing leucine-rich repeat (LRR) domains. LRR proteins contribute to, e.g. hormone signalling, receptor–ligand interactions, and immune responses (Padmanabhan et al. 2009), and have been shown to confer protection against PM in grapevine, common bean, and wheat (Goyal et al. 2020; Binagwa et al. 2021; Wang et al. 2022). Lynn et al. (2024) also identified numerous candidate genes, including those with LRR domains, associated with LPM resistance in strawberry. The *Powdery Mildew Resistant 5* (*PMR5*) gene was initially identified through a mutant screen in Arabidopsis, where mutations in this gene increased resistance to powdery mildew (Vogel et al. 2004). We found also a PMR5-related protein gene in our q.LPM.Rec-3B.2 interval.

### Possible origins and future analysis of the major QTL

According to our findings, the major QTL region on chromosome 3B is populated by multiple candidate genes for powdery mildew resistance. While different MTAs on q.LPM.Rec-3B.2 fit to contrasting modes of inheritance, ranging from dominant to additive (Fig. 5b), the region could in fact contain several resistance-associated loci in linkage disequilibrium. Further dissection of this resistance region would benefit from QTL mapping in new progenies, potentially leading to identification of haplotypes carrying multiple resistance alleles. Genome editing targeting the validated candidate genes could then be applied to obtain functional evidence of their role in PM resistance.

The literature on the original grandparental accessions of the ReC population suggests that several resistance factors could have been inherited from the *F. virginiana* parents. All four of the grandparental *F. virginiana* accessions (MR10, RH23, RH 30, and Frederick 9) were among the most resistant to PM in a two-year field trial in Florida, USA (Kennedy et al. 2013). A large two-year trial in Minnesota that evaluated progenies of *F.* × *ananassa* cultivars and *F. virginiana* accessions found progenies of MR10 on average susceptible, while those of RH23 or RH 30 were intermediate, and Frederick 9 highly resistant (Hancock et al. 2002). However, in our trials, the single progeny from Frederick 9 was not among the most resistant. Between the *F. chiloensis* grandparental accessions in the Florida trial, RCP37 was in both years diseased, but less severely than Scotts Creek and NAH 3 (Kennedy et al. 2013). Accordingly in our trial, the three progenies that included RCP37 as one of their grandparents (PPPS_03, 12 and 13) were among the most resistant against LPM. Hence, *F. chiloensis* alleles could also contribute to resistance and warrant further inspection. It is relevant to recognize that even though the genetic diversity of our ReC population is somewhat restricted due to the bottlenecks caused by the stringent selection process from various wild accessions and their subsequent mating and further selection (Hancock et al. 1993, 2010), the population combines several potential sources of powdery mildew resistance from taxonomically and geographically distinct sources (Hancock et al. 2002; Kennedy et al. 2013). Thus, our findings provide valuable insights into resistant pre-breeding germplasm resources that could be instrumental for future genome-informed breeding efforts in garden strawberry.

## Conclusions

Most of the currently available strawberry cultivars are susceptible or partially susceptible to leaf and fruit PM disease. The multi-familial ReC population, derived from various subspecies and geographical origins of *F. virginiana* and *F. chiloensis*, represents a valuable source of novel alleles for PM resistance. Using the ReC population, we identified several genetic loci with major to moderate effects on PM resistance. Notably, the association between the identified SNP markers and the underlying resistance alleles was consistent across years, GWA models, and environments. This validated several identified QTLs, particularly a novel q.LPM.Rec-3B.2 on chromosome 3B, which has relevant candidate genes in the region. Our results show that non-additive GWA can provide further insight into the manifestation of resistance alleles, thereby enhancing the ability to dissect complex traits such as PM resistance in strawberry. Future research should focus on fine mapping and functional validation of candidate genes and the incorporation of their resistance-conferring alleles into strawberry breeding programmes. The results here will support marker-assisted selection when integrating the allelic diversity from reconstructed strawberry populations into strawberry breeding programmes.

## Supplementary Information

Below is the link to the electronic supplementary material.

Supplementary datasets:
Dataset_S1: Filtered genotype data (HapMap format) and phenotype data (BLUEs) for GWA.
Dataset_S2: Trait-associated SNPs, GWA models and approaches per PM QTLs from GWA and test statistics from Bayesian multivariate analysis.
Dataset_S3: List of candidate genes on chromosome 3B. 1 (ZIP 15347 KB)

Supplementary Figures and Tables (PDF 2247 KB)

## Data Availability

The datasets analysed during the current study are available in the supplementary Dataset files (S1, S2, and S3).
