# Supplementary material for: A major QTL region associated with powdery mildew resistance in leaves and fruits of the reconstructed garden strawberry
Source: Theor Appl Genet. 2025 Apr 7;138(4):93. doi: 10.1007/s00122-025-04871-6 (PMC11976356; doi:10.1007/s00122-025-04871-6)
Supplement: Supplementary file 2 — Supplementary Figures and Tables (PDF 2247 KB) [file 122_2025_4871_MOESM2_ESM.pdf]

Supplementary Figures and Tables

Table S1. ReC population biparental progenies, pedigree, cluster info and mean±SE per trait from the field trial.

Progeny	n[1]	Pedigree of *F. chiloensis* parent[2]	Pedigree of *F. virginiana* parent[2]	DAPC cluster	Endpoint	Mean	AUDPS	AUDPC	FPM
PPPS_01	24	HM1 × CFRA 24	MR 10 × RH 23	2	1.65 ± 0.32	1.29 ± 0.22	16.54 ± 2.92	14.12 ± 2.50	1.24 ± 0.26
PPPS_02	31	RC-8	Frederick 9	1	1.78 ± 0.33	1.32 ± 0.23	17.10 ± 2.89	14.73 ± 2.49	1.12 ± 0.27
PPPS_03	10	HM1 × RCP-37	MR 10 × RH 23	3	1.64 ± 0.33	1.13 ± 0.22	14.92 ± 2.87	12.95 ± 2.56	1.74 ± 0.25
PPPS_04	26	NAH 3 × CFRA 372	RH 30 × MR 10	2	1.72 ± 0.32	1.23 ± 0.22	16.02 ± 2.81	13.78 ± 2-43	1.31 ± 0.27
PPPS_05	25	NAH 3 × CFRA 372	MR 10 × RH 23	2	1.67 ± 0.32	1.21 ± 0.22	15.85 ± 2.75	13.78 ± 2.40	1.44 ± 0.27
PPPS_06	23	NAH 3 × 2 MAR 1A	RH 30 × MR 10	2	2.29 ± 0.33	1.67 ± 0.22	21.48 ± 2.86	18.27 ± 2.45	1.31 ± 0.27
PPPS_07	26	NAH 3 × 2 MAR 1A	MR 10 × RH 23	2	1.90 ± 0.31	1.41 ± 0.22	18.18 ± 2.87	15.56 ± 2.46	1.46 ± 0.26
PPPS_08	31	CFRA 24	RH 30	4	2.04 ± 0.33	1.63 ± 0.23	20.86 ± 2.94	17.77 ± 2.50	1.34 ± 0.27
PPPS_09	28	SC × 2 BRA 1A	MR 10 × RH 23	3	1.99 ± 0.31	1.41 ± 0.21	18.33 ± 2.85	15.81 ± 2.38	1.72 ± 0.27
PPPS_10	26	SC × 2 MAR 1A	RH 30 × MR 10	3	1.96 ± 0.33	1.38 ± 0.23	17.99 ± 2.87	15.48 ± 2.56	1.47 ± 0.27
PPPS_11	32	SC × 2 MAR 1A	MR 10 × RH 23	3	1.65 ± 0.32	1.18 ± 0.23	15.46 ± 2.93	13.31 ± 2.51	1.36 ± 0.26
PPPS_12	14	SC × RCP-37	MR 10 × RH 23	3	1.10 ± 0.32	0.72 ± 0.22	9.68 ± 2.81	8.40 ± 2.42	1.42 ± 0.24
PPPS_13	23	SC × RCP-37	RH 30 × MR 10	3	1.28 ± 0.32	0.88 ± 0.23	11.59 ± 2-87	10.06 ± 2-51	1.31 ± 0.26

[1] 'n' indicates the number of individuals per progeny.

[2] The U.S. National Plant Germplasm System (NGPS) accession identifiers: *F. chiloensis* sp. *pacifica*: SC, PI 612490; *F. chiloensis* sp. *lucida*: HM1, PI 612489; RCP-37, PI 551445; *F. chiloensis* sp. *chiloensis*: NAH 3, PI 612318; 2 MAR 1A, PI 602567; 2 BRA 1A, PI 612316; *F. chiloensis* with mixed ancestry: CFRA 24, PI 236579; RC-8, JH-101, a complex hybrid accession (Sauble Beach 8 x Del Norte) x (Lions Head 3 x Del Norte); CFRA 372, PI 551736; *F. virginiana* sp. *virginiana*: MR 10, PI 612497; F 9, PI 612493; RH 23, PI 612498; RH 30, PI 612499.

Table S2. Analysis of Variance results for all four LPM aggregates across three years of field experiment. Significant differences at ($p < 0.01$) represented as **.

Aggregate	Factors	DF	Sum of Squares	Significance
**Endpoint**	Genotype	325	1,616.024	**
	Year	2	984.499	**
	Genotype x Year	604	450.729	**
	Residuals	931	275.757	
**Mean**	Genotype	325	900.715	**
	Year	2	593.698	**
	Genotype x Year	604	233.340	**
	Residuals	931	142.503	
**AUDPC**	Genotype	325	105,650.87	**
	Year	2	96,059.49	**
	Genotype x Year	604	26,983.10	**
	Residuals	931	17,880.76	
**AUDPS**	Genotype	325	143,890.13	**
	Year	2	124,940.04	**
	Genotype x Year	604	36,223.31	**
	Residuals	931	23,430.74	

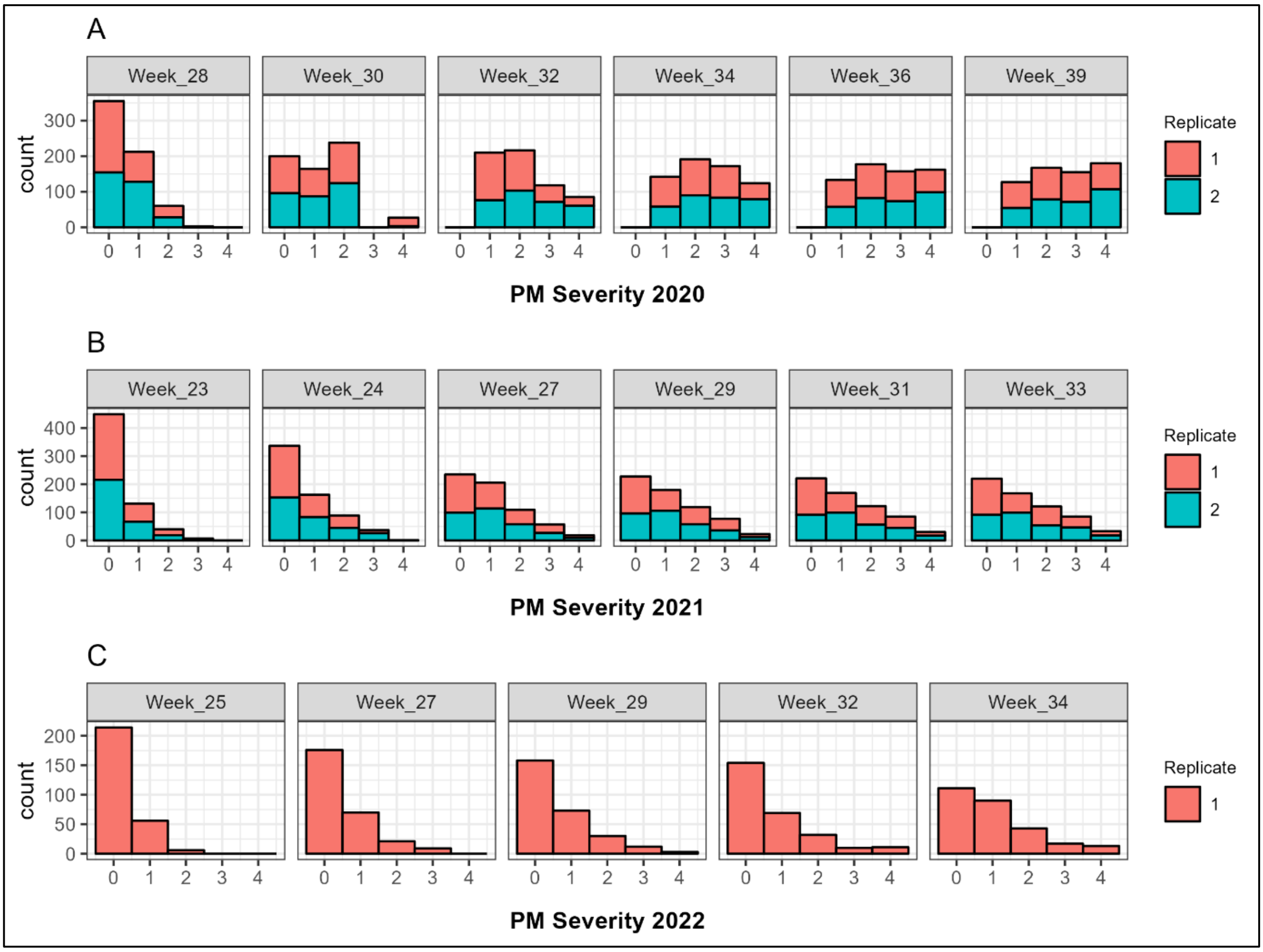

Figure S1. Progression of LPM scores for the ReC population in the field experiment. The week number represents the corresponding observation time for the respective year.

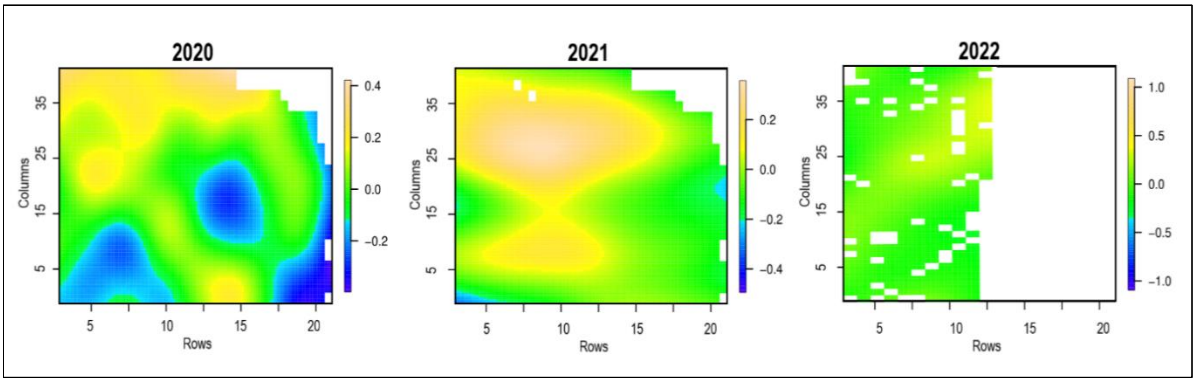

Figure S2. Spatial trends of leaf powdery mildew disease symptoms across plots in the Rec field trial. The scale bar indicates the relative level of disease incidence from blue (low) to orange (high). In 2022, only the first replicate was observed.

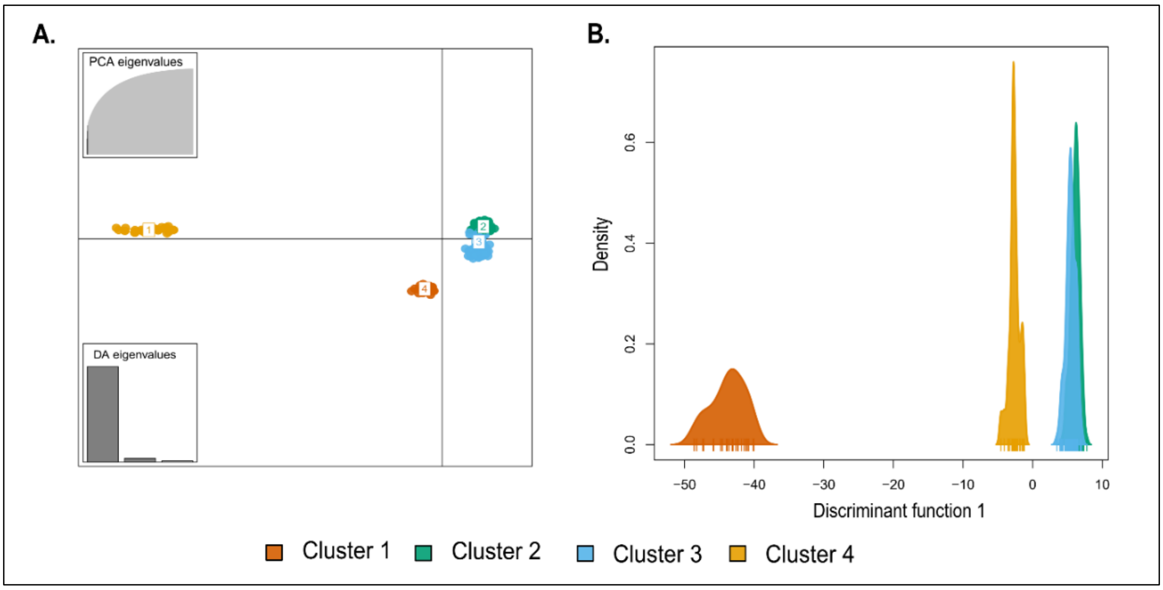

Figure S3. Discriminant Analysis of Principal Components (DAPC) scatter plot (A) and individual density plot on the first discriminant function (B) of 298 ReC genotypes using 20779 SNP markers. Identified clusters are coloured as per the legend on the bottom.

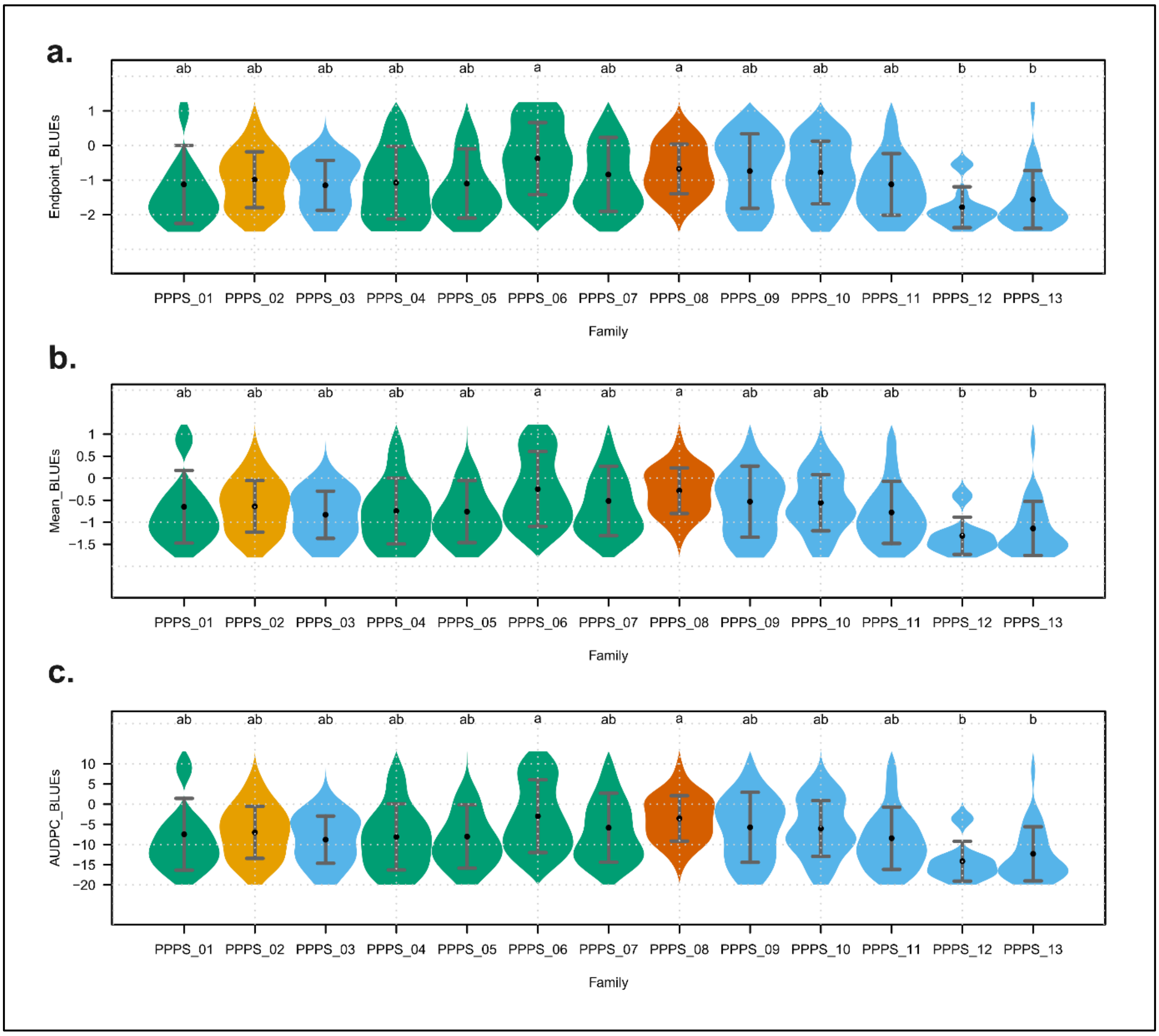

Figure S4. The family-wise distributions of the leaf powdery mildew aggregates Endpoint (a), Mean (b), and AUDPC BLUEs (c) of the 13 families of the ReC population. Grey whiskers represent the standard deviation, while black dots represent the mean value per family. Different colours of the violins correspond to progeny clusters based on discriminant analysis of principal components. Different letters on violin plots indicate significant differences based on Tukey HSD ($p < 0.05$).

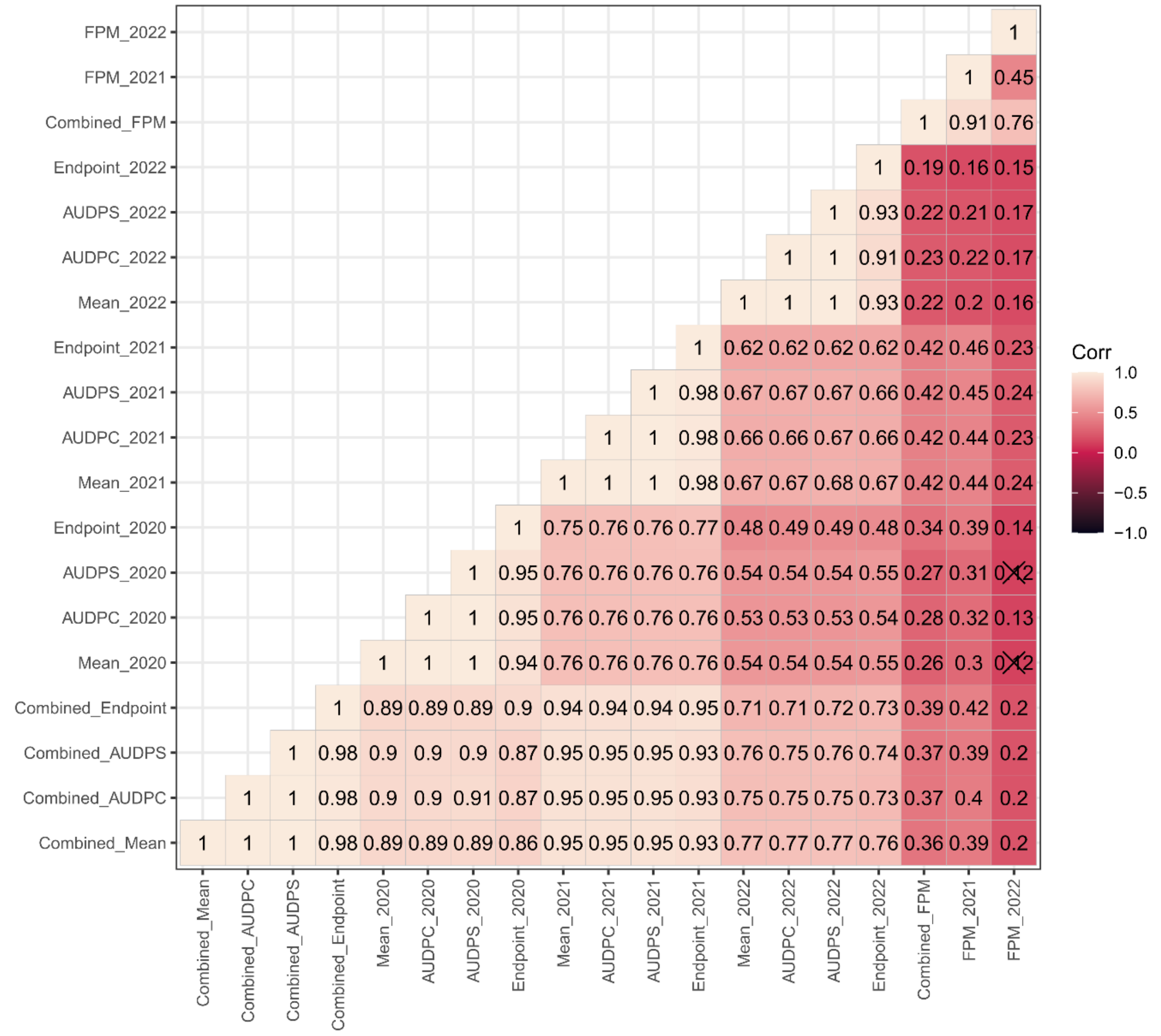

Figure S5. Correlation between all LPM aggregates and FPM scores for both combined and year-wise datasets. The correlation coefficients (r) are presented, with non-significant values marked with a strikethrough (*p < 0.01*).

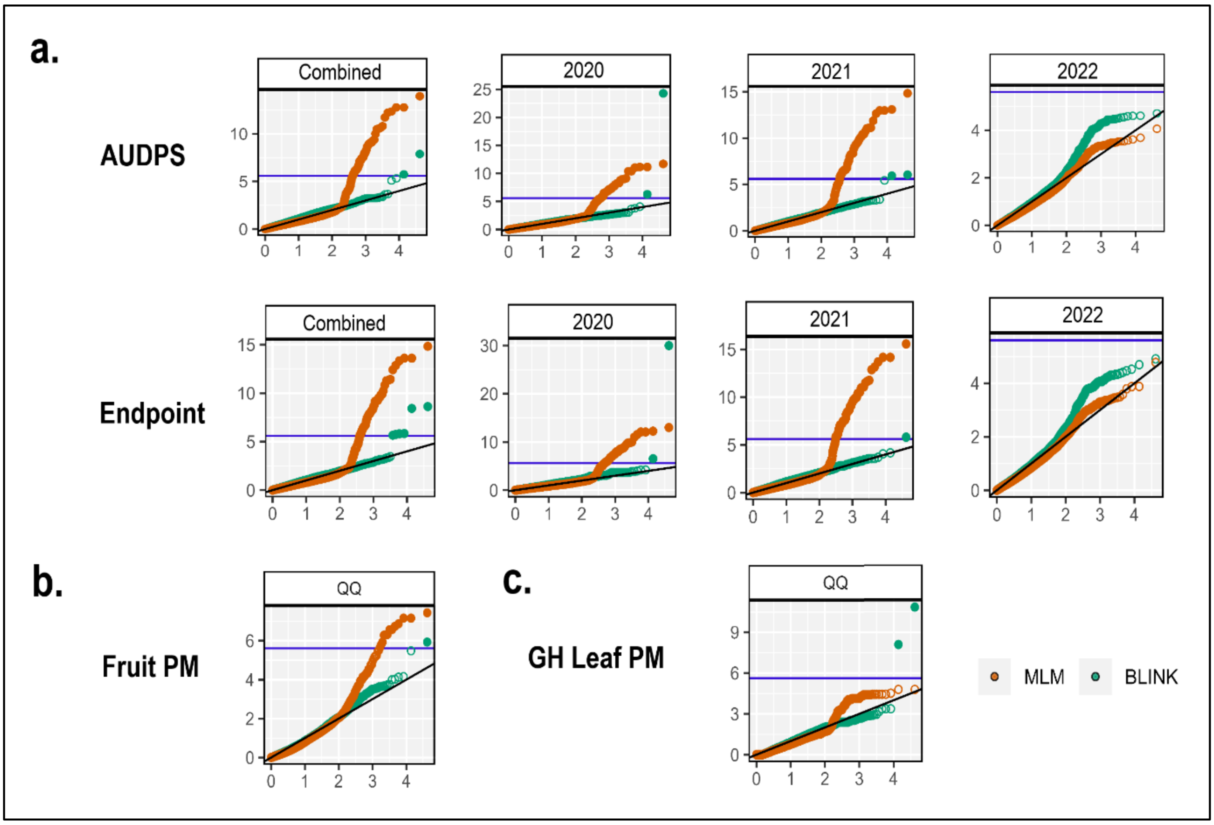

Figure S6. GWAS QQ plots for all the studied parameters for leaf powdery mildew (a), fruit powdery mildew (b), and greenhouse leaf powdery mildew (c). For each QQ-plot, the x-axis represents the expected -log(p) values and y-axis represents the observed -log(p) values for each model. The black line represents the expected distribution while the blue line indicates the Bonferroni (−log10(p) = 5.61) threshold. All models effectively controlled for inflation risk, as evidenced by the alignment of the observed values with the expected distribution, indicating reliable results across the GWAS analyses.

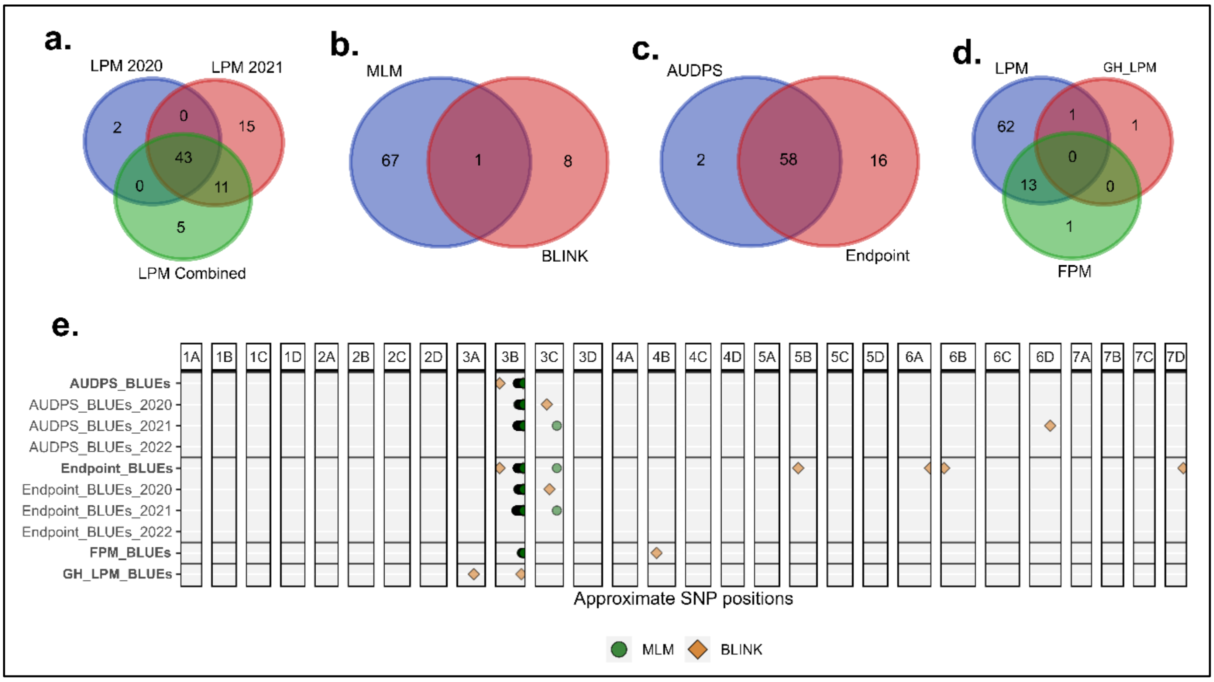

Figure S7. Common and unique MTAs at the Bonferroni threshold (−log10(p) = 5.61) for leaf powdery mildew (LPM) across years (a), across GWAS models (b), across aggregates for three years combined analysis (c), and between field, greenhouse (GH) and fruit powdery mildew (FPM) (d), shown as Venn diagrams. The summary diagram (e) represents the genomic locations of MTAs identified by MLM and BLINK models.

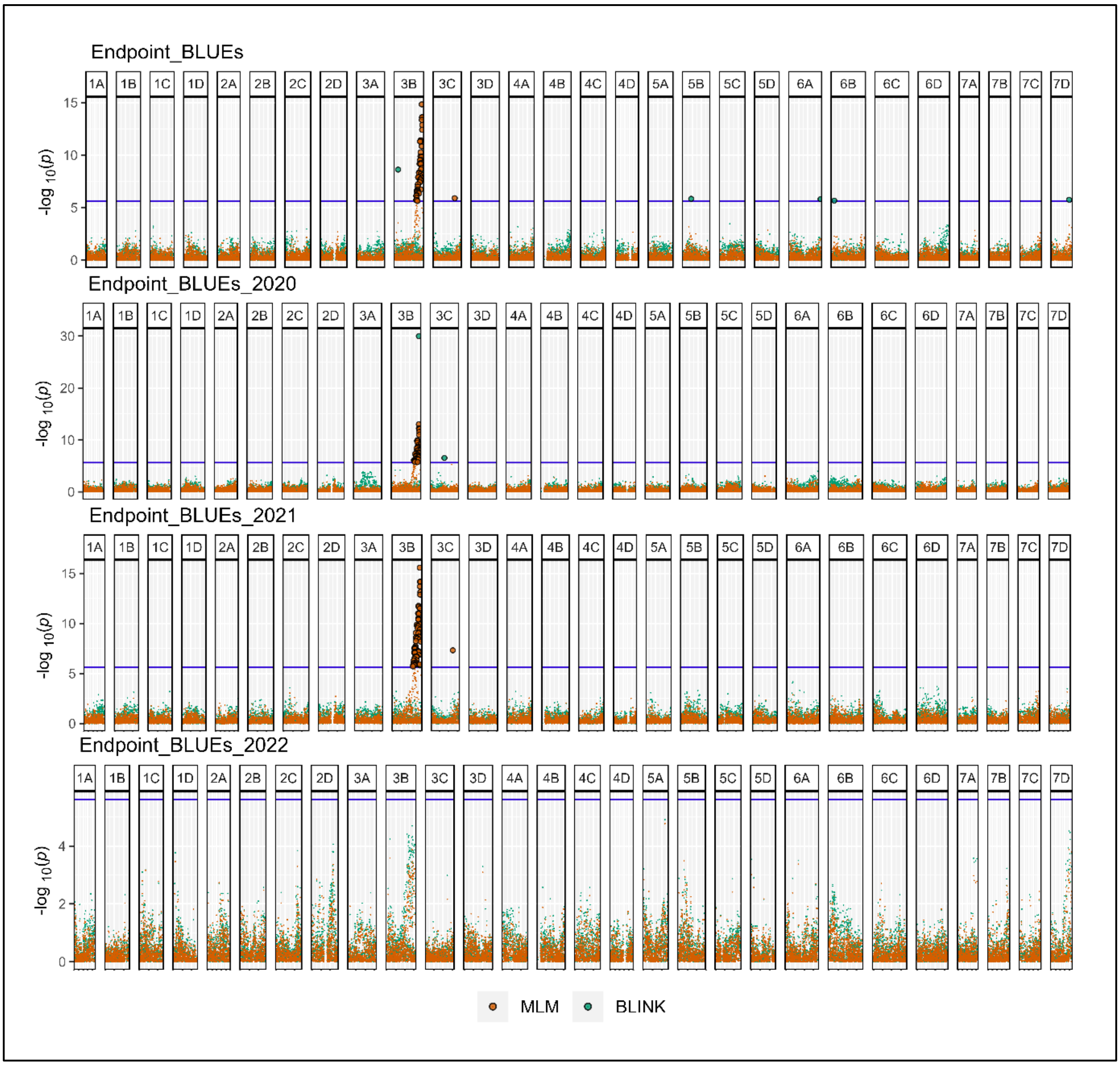

Figure S8. Stacked Manhattan plots for Endpoint year-wise and combined-year analysis. Significant SNPs are highlighted and coloured according to the respective GWAS model. The blue line indicates the Bonferroni (−log10(p) = 5.61) threshold.

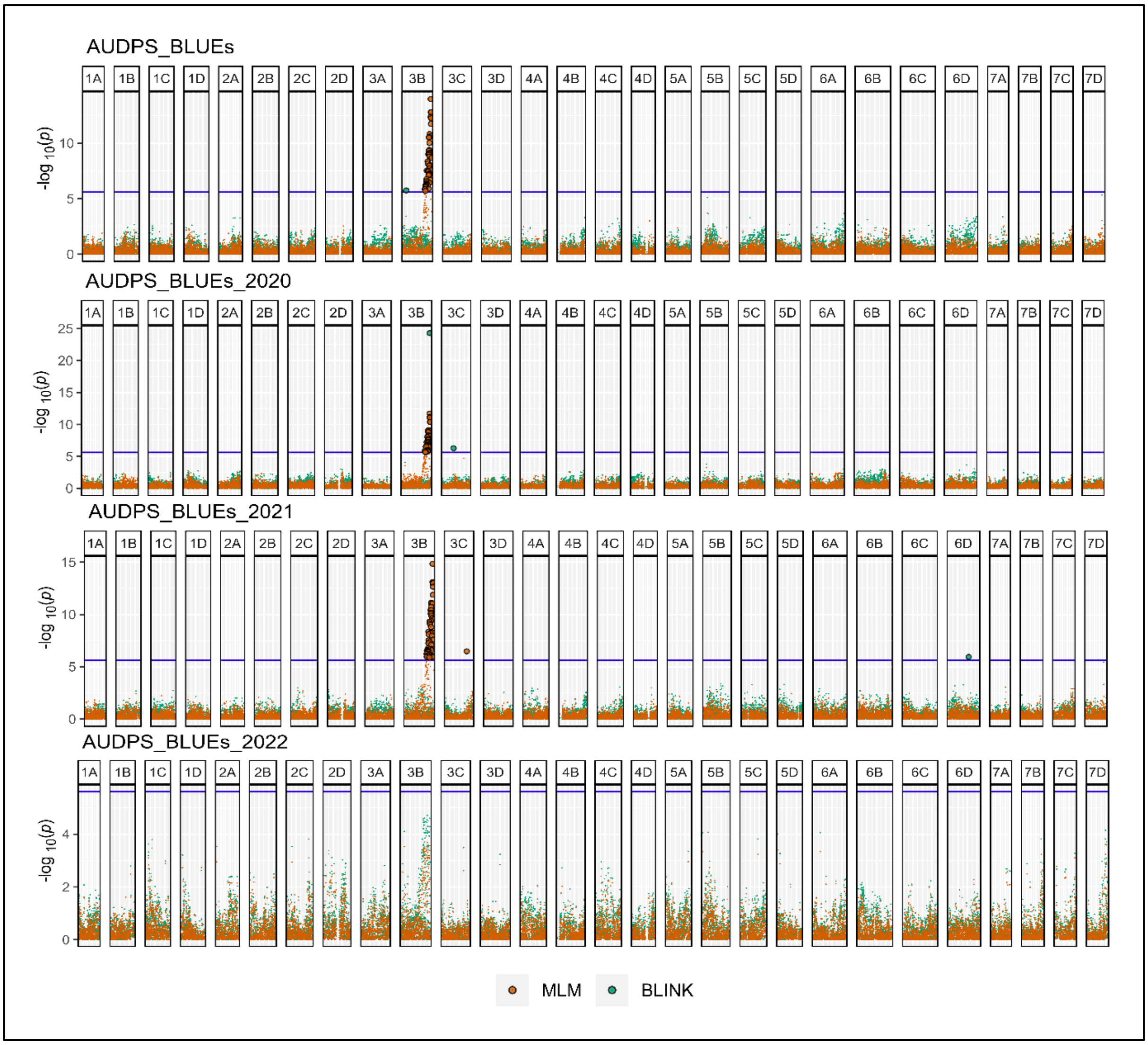

Figure S9. Stacked Manhattan plots for AUDPS year-wise and combined-year analysis. Significant SNPs are highlighted and coloured according to the respective GWAS model. Blue line indicates the Bonferroni (−log10(p) = 5.61) threshold.

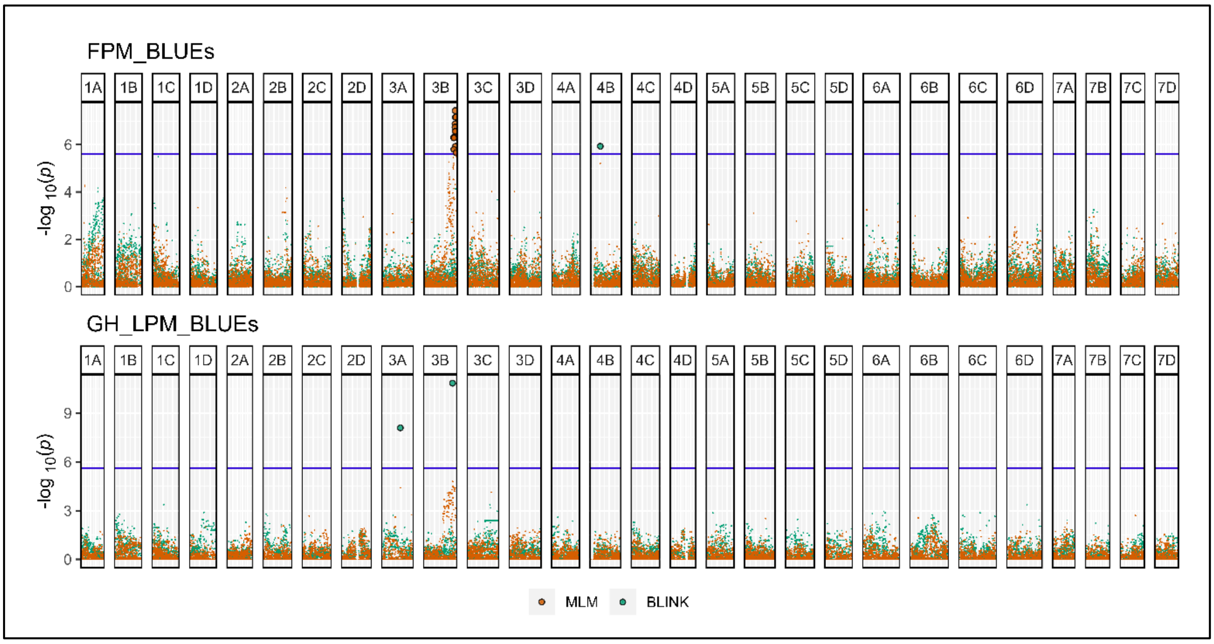

Figure S10. Stacked Manhattan plots for FPM (upper) and greenhouse LPM (lower). Significant SNPs are highlighted and coloured according to the respective GWAS model. The blue line indicates the Bonferroni (−log10(p) = 5.61) threshold.

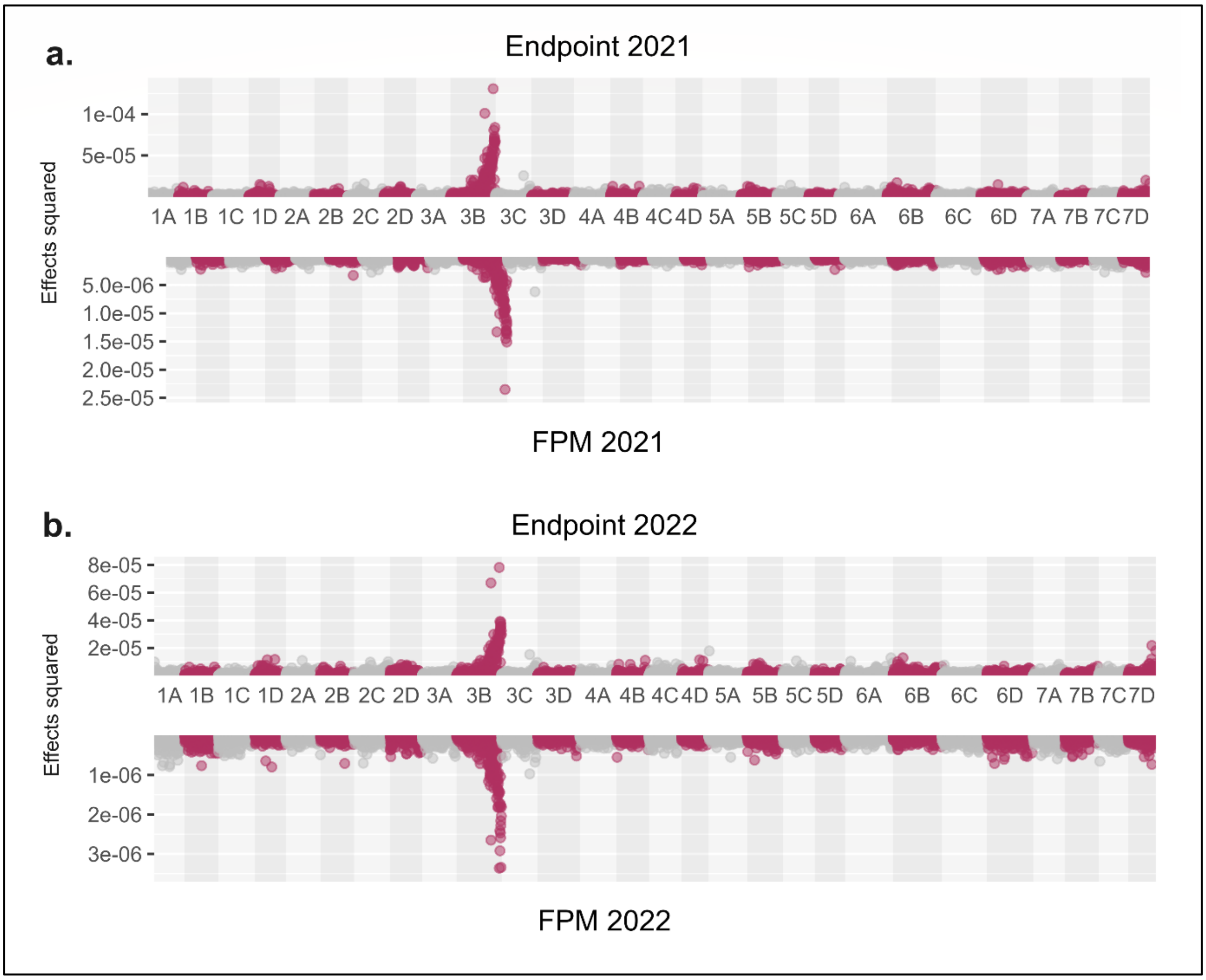

Figure S11. SNP effects from Bayesian multi-trait modelling using trait data from 2021 (a) and 2022 (b) jointly in the same four-trait model. The y-axes of the Miami plots show squared effects per Endpoint and FPM for year 2021 (a) and 2022 (b).

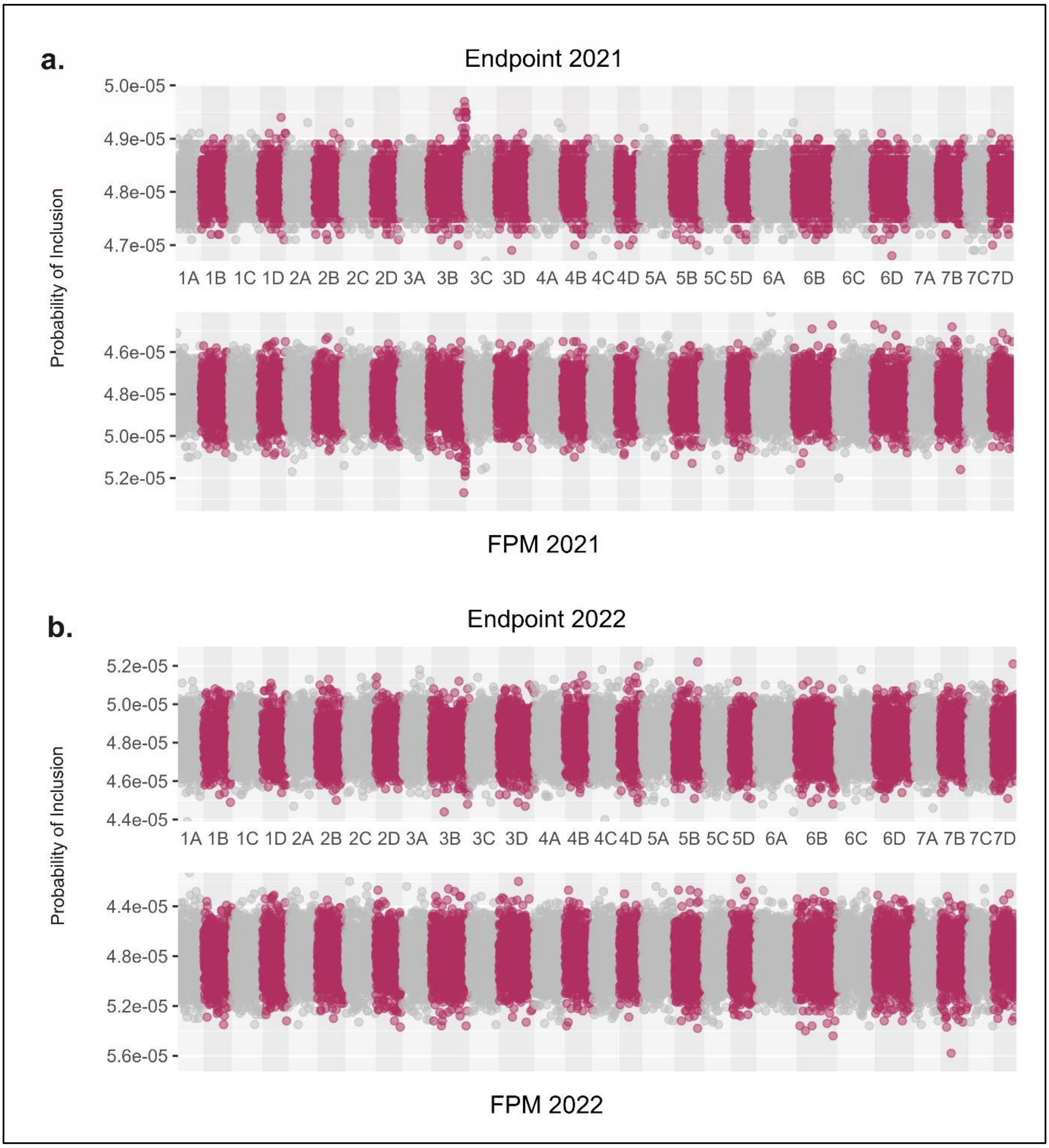

Figure S12. Probability of inclusion for SNP markers from Bayesian multi-trait modelling using trait data from 2021 (a) and 2022 (b) jointly in the same four-trait model. The upper plot in both panes shows the Endpoint and the lower the Fruit PM specific values per year. The y-axis shows the posterior probability of inclusion per trait per year.

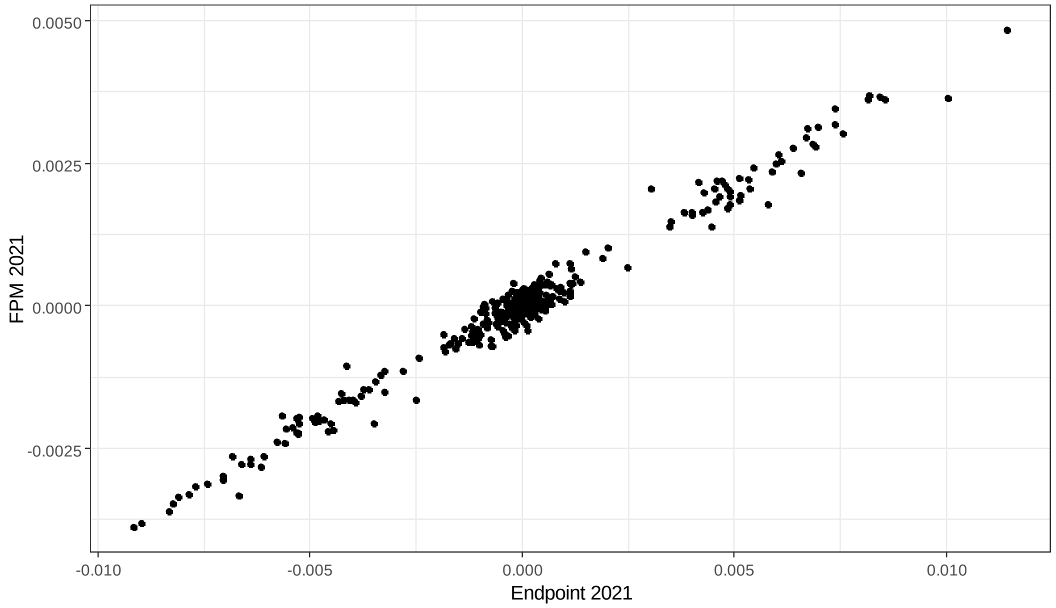

Figure S13. Effect sizes of the SNPs of the 3B QTL region q.LPM.Rec-3B.2 on fruit and leaf PM for the year 2021 extracted from the Bayesian multi-trait modelling results.

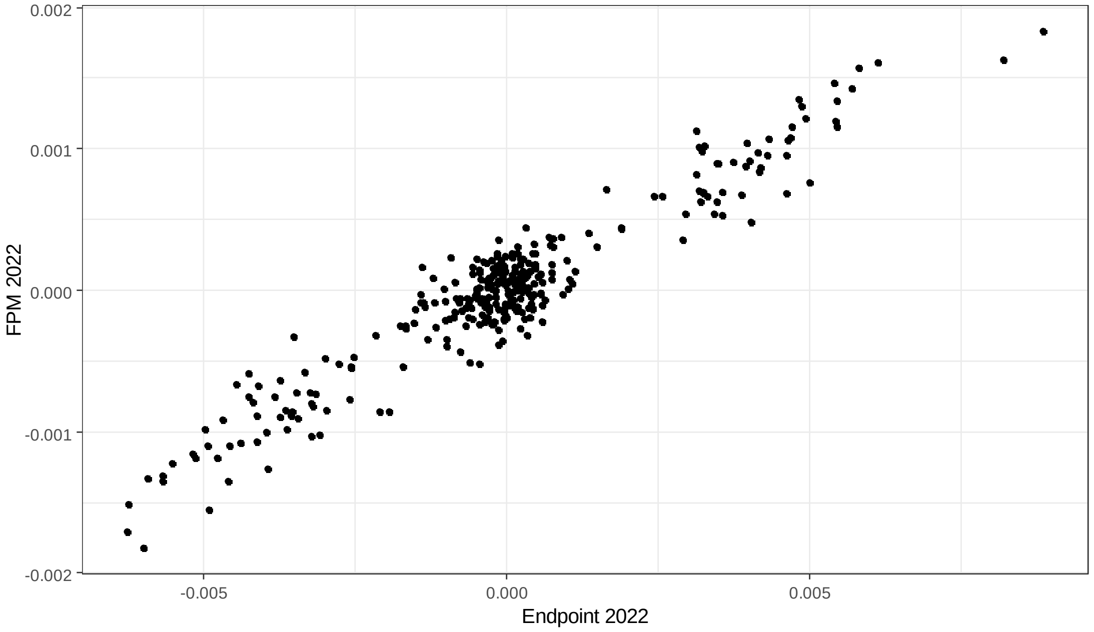

Figure S14. Effect sizes of the SNPs of the 3B QTL region q.LPM.Rec-3B.2 on fruit and leaf PM for the year 2022 extracted from the Bayesian multi-trait modelling results.
